# Smad2 and Smad3 Regulate Chondrocyte Proliferation and Differentiation in the Growth Plate

**DOI:** 10.1371/journal.pgen.1006352

**Published:** 2016-10-14

**Authors:** Weiguang Wang, Buer Song, Teni Anbarchian, Anna Shirazyan, Joshua E. Sadik, Karen M. Lyons

**Affiliations:** 1 Department of Orthopaedic Surgery, David Geffen School of Medicine, University of California, Los Angeles, Los Angeles, California, United States of America; 2 Department of Molecular, Cell and Developmental Biology, University of California, Los Angeles, Los Angeles, California, United States of America; Harvard School of Dental Medicine, UNITED STATES

## Abstract

TGFβs act through canonical and non-canonical pathways, and canonical signals are transduced via Smad2 and Smad3. However, the contribution of canonical vs. non-canonical pathways in cartilage is unknown because the role of Smad2 in chondrogenesis has not been investigated *in vivo*. Therefore, we analyzed mice in which Smad2 is deleted in cartilage (*Smad2*^*CKO*^), global *Smad3*^*-/-*^ mutants, and crosses of these strains. Growth plates at birth from all mutant strains exhibited expanded columnar and hypertrophic zones, linked to increased proliferation in resting chondrocytes. Defects were more severe in *Smad2*^*CKO*^ and *Smad2*^*CKO*^*;Smad3*^-/-^ *(Smad2/3)* mutant mice than in *Smad3*^*-/-*^ mice, demonstrating that Smad2 plays a role in chondrogenesis. Increased levels of *Ihh* RNA, a key regulator of chondrocyte proliferation and differentiation, were seen in prehypertrophic chondrocytes in the three mutant strains at birth. In accordance, TGFβ treatment decreased *Ihh* RNA levels in primary chondrocytes from control (*Smad2*^*fx/fx*^) mice, but inhibition was impaired in cells from mutants. Consistent with the skeletal phenotype, the impact on TGFβ-mediated inhibition of *Ihh* RNA expression was more severe in *Smad2*^*CKO*^ than in *Smad3*^*-/-*^ cells. Putative Smad2/3 binding elements (SBEs) were identified in the proximal *Ihh* promoter. Mutagenesis demonstrated a role for three of them. ChIP analysis suggested that Smad2 and Smad3 have different affinities for these SBEs, and that the repressors SnoN and Ski were differentially recruited by Smad2 and Smad3, respectively. Furthermore, nuclear localization of the repressor Hdac4 was decreased in growth plates of *Smad2*^*CKO*^ and double mutant mice. TGFβ induced association of Hdac4 with Smad2, but not with Smad3, on the *Ihh* promoter. Overall, these studies revealed that Smad2 plays an essential role in the development of the growth plate, that both Smads 2 and 3 inhibit *Ihh* expression in the neonatal growth plate, and suggested they accomplish this by binding to distinct SBEs, mediating assembly of distinct repressive complexes.

## Author Summary

The cartilage growth plate regulates the size and shape of nearly every skeletal element in the body. TGFβs are potent inducers of cartilage formation, but the mechanisms by which they transduce their signals in cartilage during development are poorly understood. Similarly, there is strong evidence that dysregulation of the TGFβ pathway increases the risk for osteoarthritis (OA) in humans, but the underlying mechanisms are unknown. TGFβs transduce their signals through a canonical pathway involving Smad2 and Smad3, and through several non-canonical pathways. However, the roles of canonical vs. noncanonical signaling are unknown in cartilage because the combined roles of Smad2 and Smad3 have not been determined. We generated mice lacking both Smad2 and Smad3 in cartilage in order to determine the role of canonical TGFβ signaling during embryonic development. We determined that Smad2 has a more prominent role than Smad3 in non-hypertrophic chondrocytes in the growth plate, and identified elevated levels of *Ihh* RNA in neonatal cartilage in *Smad2* and *Smad3* mutants. These findings may be important because Ihh is a vital regulator of cartilage proliferation and differentiation during cartilage development. More generally, the studies identify how Smad2 and Smad3 can regulate a common target gene through distinct mechanisms.

## Introduction

The cartilage growth plate is the primary driver of endochondral bone growth. In the growth plate, resting, columnar, prehypertrophic and hypertrophic chondrocytes are arrayed in discrete zones. Resting chondrocytes, located at the top of the growth plate, are small and relatively quiescent. Upon stimulation by extracellular signals, cells near the bottom of the resting zone transition to columnar chondrocytes, which exhibit a higher rate of proliferation and a flatter morphology. These cells form stacks along the long axis of the developing skeletal element. Columnar cells at the bottom of this zone eventually exit the mitotic phase and become prehypertrophic chondrocytes. Prehypertrophic cells further differentiate into enlarged hypertrophic cells, comprising a zone adjacent to the site of replacement of cartilage by bone. Chondrocyte proliferation and differentiation in the growth plate is tightly regulated by Indian hedgehog (Ihh) and parathyroid hormone-related peptide (Pthrp). Ihh, a secreted protein expressed in prehypertrophic chondrocytes, stimulates cell proliferation and differentiation. Its role in proliferation is mediated in part by inducing Pthrp expression in epiphyseal resting chondrocytes. Secreted Pthrp maintains columnar cells in a mitotic state, preventing their transition to the pre-hypertrophic phase, and hence negatively regulating Ihh expression. Once cells escape the zone of influence of Pthrp, they exit the cell cycle, become prehypertrophic, and upregulate Ihh expression, which promotes hypertrophy and matrix mineralization. This feedback loop thus controls the transition of chondrocytes through each zone of the growth plate.

Transforming growth factor βs (TGFβs) and activins are secreted proteins that are members of the TGFβ superfamily of growth factors. TGFβs and activins bind to distinct receptor complexes, but activate similar signal transduction pathways. Binding of TGFβs or activins to their receptors leads to activation of the kinase activity of the receptor. The activated receptor complexes then transduce signals through multple pathways. These pathways can be broadly divided into Smad-dependent and Smad-independent pathways [1–3]. In the canonical Smad-dependent pathway, activated receptor complexes phosphorylate the receptor-activated Smads (R-Smads), Smad2 and Smad3. Smads 2 and 3 are transcription factors; once phosphorylated, they form hetero-oligomeric complexes with the transcription factor Smad4. These complexes enter the nucleus, bind promoters, and regulate target gene expression. In addition, there exist numerous non-canonical Smad-independent pathways for transduction of TGFβ signals, such as MAP kinases, RhoA and mTOR [4–8].

TGFβs play critical roles in growth plate chondrocyte proliferation and differentiation, and in the maintenance of articular chondrocytes [9–17]. There are three TGFβ isoforms in mammals: TGFβ1, 2, 3. Only *Tgfb2* mutants exhibit obvious skeletal dysplasia. *Tgfb2*^*-/-*^ mice exhibit embryonic lethality, accompanied by skeletal defects that include shortened limbs, axial, and craniofacial defects [18]. The role of the TGFβ signaling pathway in cartilage has been studied most extensively using mice lacking the type II TGFβ receptor TGFβRII. This receptor is required for transduction of the TGFβ pathway by TGFβs 1–3, but is not used by activin ligands. *Tgfbr2;Prrx1Cre* mice, in which TGFβRII is ablated in limb bud mesenchyme, exhibit growth plates with decreased proliferation and accelerated onset of hypertrophy, but delayed terminal maturation [19, 20]. In contrast, conditional deletion of the TGFβRII in committed *Col2a1*-expressing chondrocytes did not lead to obvious defects in appendicular elements [21]. These findings suggest that TGFβRII transduces TGFβ signals at prechondrogenic stages and/or in perichondrium, but may not have a substantial role in cartilage once a growth plate forms.

The extent to which Smad2/3-dependent signaling mediated by TGFβ and activins is required in developing cartilage is unknown. Smad2, Smad3 and Smad4 are co-expressed throughout the growth plate [12, 22–24]. Smad2, 3 and 4 are all present in articular cartilage [12, 25]. *Smad3*^*-/-*^ mice are born with a normal skeletal phenotype, but develop postnatal dwarfism and osteoarthritis-like pathologies in adulthood [12, 26–28].

The function of Smad2 in cartilage during embryogenesis has not been characterized. Smad2 and Smad3 have some distinct roles in mediating TGFβ/activin signaling. Smad3 can bind DNA directly, whereas Smad2 regulates gene expression by interacting with Smad3 or other transcription factors [29]. Mice lacking *Smad2* die before 8.5 days of development (E8.5) [30], precluding a genetic analysis of its function in chondrogenesis. It is not known whether Smad2 partially compensates for the loss of Smad3 in the growth plates of *Smad3*^*-/-*^ mice. Studies in *Smad2*^*-/-*^ vs. *Smad3*^*-/-*^ epiblast, epithelial cells and fibroblasts show that Smads 2 and 3 regulate some common and some distinct target genes [31, 32]. Overexpression of Smad2 or Smad3 can block the spontaneous maturation observed in *Smad3*-deficient chondrocytes [33], providing support for the hypothesis that Smad2 and Smad3 may have some overlapping functions in the growth plate.

To define the role of Smad2/3-mediated signaling in cartilage, we generated mice with conditional deletion of *Smad2* using *Col2a1-Cre* (*Smad2*^*fx/fx*^*;Col2Cre*, hereafter referred to as *Smad2*^*CKO*^), mice with *Smad3* globally deleted (*Smad3*^*-/-*^), and the corresponding double mutants (*Smad2/3*). Loss of *Smad2* leads to a growth plate phenotype in neonates that is more severe than that seen in *Smad3*^*-/-*^ mice. At the neonatal stage, *Smad2/3 (Smad2*^*CKO*^*;Smad3*^*-/-*^*)* double mutants exhibit more severe defects in the hypertrophic zone than do *Smad2*^*CKO*^ or *Smad3*^*-/-*^ mice. Defects include elevated levels of proliferation in resting zone chondrocytes at neonatal stages, leading to enlarged columnar and hypertrophic zones. This may be a consequence of depletion of the resting zone due to the accelerated entry of resting zone chondrocytes into the columnar zone. Overall, our results suggest that Smad2 inhibits proliferation of resting zone chondrocytes during embryogenesis, and acts as a negative regulator of *Ihh* expression, and that Smad2 and Smad3 have some overlapping functions in cartilage. Our results show that both of these Smads are required at neonatal stages for normal chondrocyte proliferation and differentiation in the growth plates.

## Results

### Smad2 and Smad3 are activated throughout the growth plate

We generated mice lacking Smad2 and Smad3 in cartilage in order to study the role of canonical Smad2/3-mediated signaling in chondrogenesis. *Smad2*^*fx/fx*^ mice were intercrossed with *Col2a1Cre* mice to generate *Smad2*^*fx/fx*^*;Col2a1Cre* (*Smad2*^*CKO*^) mice. *Smad2*^*CKO*^ mice are viable and fertile. The *Smad3*^*-/-*^ allele we used has a LacZ cassette and internal ribosomal entry site (IRES) inserted in the second exon, leading to a loss-of-function allele [27]. We crossed *Smad3*^*+/-*^ mice with *Smad2*^*CKO*^ mice to generate *Smad2*^*fx/fx*^*;Col2a1Cre;Smad3*^*-/-*^ (*Smad2/3* double mutant) mice.

To confirm efficient Cre-mediated excision, levels of activated C-terminal phosphorylated Smad2 and Smad3 were examined by IHC using an antibody that recognizes both pSmad2 and pSmad3 (S1 Fig). No pSmad2/3 was detected in E16.5 or P0 double mutant growth plates, verifying efficient loss of Smads 2 and 3 (S1D and S1H Fig). Consistent with previous studies [34], at E16.5 and P0, pSmad2/3 is present throughout the resting, columnar, and prehypertrophic zones of the E16.5 *Smad2*^*fx/fx*^ control growth plate, and this pattern of expression persists at least until birth (S1A and S1E Fig). No obvious differences were noted in the distribution of pSmad3 in *Smad2*^*CKO*^ or pSmad2 in *Smad3*^*-/-*^ mice compared with control *Smad2*^*fx/fx*^ littermates (S1B, S1C, S1F and S1G Fig). These findings confirm previous reports that pSmads 2 and 3 have extensively overlapping distributions in the growth plate [12, 22–24].

### *Smad2*^*CKO*^*;Smad3*^*-/-*^ mutant mice exhibit subtle defects at birth followed by postnatal dwarfism

**Fig 1:**
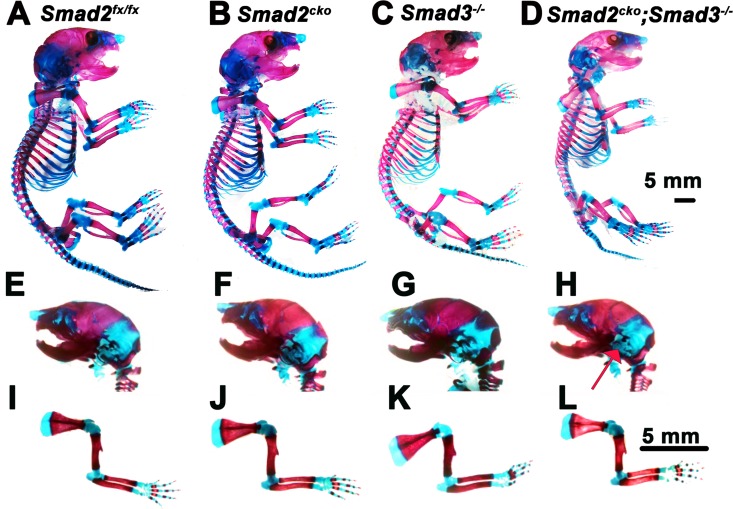
Subtle skeletal defects in *Smad2*^*CKO*^*;Smad3*^*-/-*^ mutants at P0. **(A-D)** Side views of skeletons. **(E-H)** Lateral views of skulls. Reduced ossification of the bones of the inner ear in double mutants is highlighted by arrow in (H). **(I-L)** Lateral views of forelimbs, showing no apparent differences in mutants. *Smad2*^*CKO*^ *= Smad2*^*fx/fx*^*;Col2a1-Cre*.

Consistent with previous studies [12, 26, 27], skeletal preparations revealed no apparent skeletal defects in *Smad3*^*-/-*^ mice at P0. Analysis of P0 *Smad2*^*CKO*^ mice also revealed no obvious defects (Fig 1A–1C). However, *Smad2*^*CKO*^*;Smad3*^*-/-*^ (*Smad2/3*) double mutant mice exhibited subtle defects in axial and craniofacial elements (Fig 1A and 1D). Lateral views of P0 skulls revealed reduced ossification of the bones of the inner ear in double mutants (Fig 1E–1H). The length of the body (from top of the skull to the proximal end of the tail) of double mutants is about 9.7% shorter than that of control *Smad2*^*fx/fx*^ littermates (n = 4, P = 0.04) (Figs 1A, 1D and S2A). Ventral views revealed that double mutants have slightly smaller skulls, with a shorter nasal to occipital length (94% of control (*Smad2*^*fx/fx*^), n = 3, p = 0.05), narrower cranial base (93% of control, n = 3, p = 0.04), and reduced ossification of the occipital condyle (arrow, S2B Fig).

Dorsal views of the rib cage revealed no obvious differences between *Smad2*^*CKO*^ or *Smad3*^*-/-*^ mice compared with control *Smad2*^*fx/fx*^ littermates (S2C Fig). However, double mutants have a shorter sternum and a bifurcated xiphoid process (S2C Fig). Moreover, localized defects were seen in the vertebral columns of *Smad2/3* mutants. Ventral views of the lumbar spine revealed shorter vertebrae and smaller ossified vertebral bodies in double mutants; reduced ossification was not observed in *Smad2*^*CKO*^ or *Smad3*^*-/-*^ mice (S2D Fig). No differences were evident in the cervical vertebrae in *Smad3*^*-/-*^ mice compared to control *Smad2*^*fx/fx*^ mice, but the ossification center of the axis (C2) was reduced in *Smad2*^*CKO*^ mice and in double mutants (S2E Fig). Cleared skeletal preparations revealed no clear differences in appendicular elements in *Smad2*^*CKO*^, *Smad3*^*-/-*^ mice, or *Smad2/3* double mutants (Figs 1I–1L and S2F).

As discussed above, *Smad2/3* double mutants had only subtle skeletal alterations at birth. However, these mice developed progressive postnatal dwarfism, which was not seen in *Smad2*^*CKO*^ and *Smad3*^*-/-*^ mice (S3 Fig). At 1 month, double mutants were 12.5% (n = 3, p = 0.05) shorter than control *Smad2*^*fx/fx*^ littermates (S3C Fig). These data indicate that Smad2 and Smad3 have compensatory roles in the regulation of axial skeletal growth after birth.

### Increased neonatal chondrocyte proliferation and hypertrophy in *Smad2* and *Smad3* mutant growth plates

**Fig 2:**
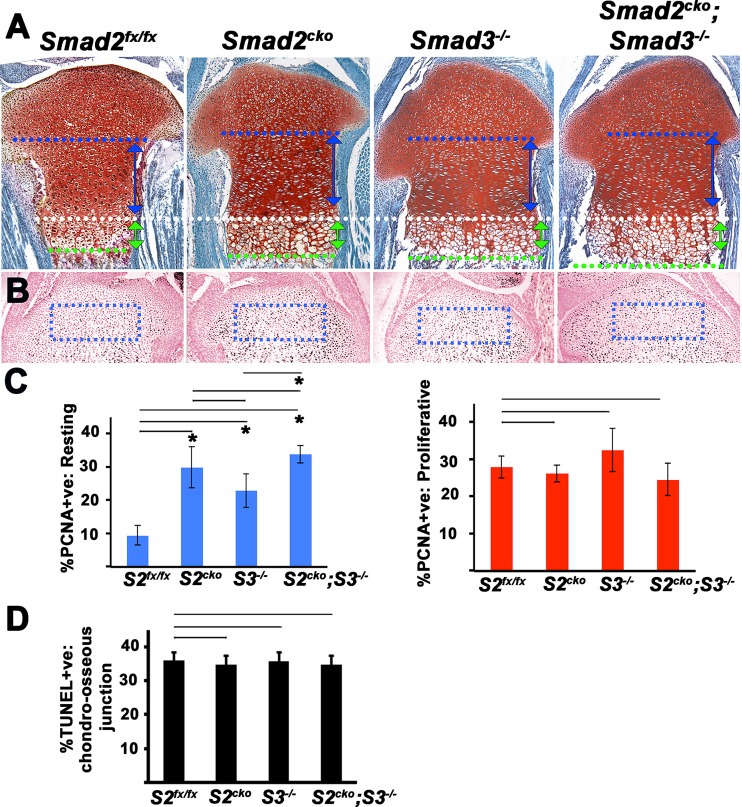
Elevated proliferation in *Smad2*^*CKO*^ and *Smad3*^*-/-*^ growth plates. All images are P0 proximal tibias. **(A)** Sections stained with safranin O. Heights of distinct growth plate zones are indicated by double arrows and demarcated by dashed lines. White dashed line is the demarcation between the columnar and prehypertrophic zones. All images are aligned to this boundary. Blue dashed lines demarcate the approximate boundary between the resting and columnar zones. Green dashed lines demarcate the boundary between the hypertrophic zone and zone of ossification. Blue double-headed arrows indicate the extent of the proliferative zone in the control *Smad2*^*fx/fx*^ growth plate. Green double-headed arrows demarcate the extent of the control *Smad2*^*fx/fx*^ hypertrophic zone. The double-headed arrows are superimposed on the images of *Smad2*^*CKO*^, *Smad3*^*-/-*^ and *Smad2*^*CKO*^*;Smad3*^*-/-*^ mutants to clarify the differences in lengths of these zones compared with control *Smad2*^*fx/fx*^. 3 litters containing mice of all of the genotypes shown in the figures were examined. At least 5 mice per genotype were examined. The images shown are from littermates. **(B)** PCNA immunostaining. All mice are littermates. **(C)** Quantitation of the percentage of PCNA-positive cells in distinct zones. The percentage of PCNA-positive cells in the resting zone (blue bars) and columnar zone (red bars) were quantified. Data are expressed as percent PCNA-positive + SE (n = 5). Asterisk, p < 0.05 compared with control *Smad2*^*fx/fx*^. **(D)** Quantitation of the percentage of TUNEL-positive cells in chondro-osseous junctions. Data are expressed as percent TUNEL-positive + SE (n = 3). There are no significant differences between *Smad2*^*CKO*^, *Smad3*^*-/-*^ and *Smad2*^*CKO*^*;Smad3*^*-/-*^ and control *Smad2*^*fx/fx*^ mice. *S2*^*fx/fx*^ = *Smad2*^*fx/fx*^. *S2*^*CKO*^ = *Smad2*^*fx/fx*^*;Col2a1Cre*. *S3*^*-/-*^ = *Smad3*^*-/-*^. *S2*^*CKO*^;*S3*^*-/-*^ = *Smad2*^*fx/fx*^*;Col2a1Cre;Smad3*^*-/-*^.

The above analysis revealed no clear impact of loss of *Smad2* or *Smad3* on appendicular length at P0. A previous study demonstrated that loss of *Smad3* led to appendicular defects beginning in the early postnatal period [12]. However, we used a different *Smad3* allele. We therefore performed a histological analysis of P0 appendicular cartilage to investigate whether the *Smad3* mutant allele we used [27] exhibits a similar phenotype. We also examined whether loss of *Smad2* exerts any effect on growth plate architecture. This analysis revealed that the lengths of the resting zones in *Smad2*^*CKO*^ and *Smad2/3* double mutants were shorter than in control *Smad2*^*fx/fx*^ mice. In contrast, the columnar and hypertrophic zones of *Smad2*^*CKO*^, *Smad3*^*-/-*^, and *Smad2/3* double mutant mice were longer than those of control *Smad2*^*fx/fx*^ littermates (Figs 2A and S4). Although both *Smad2*^*CKO*^ and *Smad3*^*-/-*^ mice exhibited elongated columnar zones, the effect was greater in *Smad2*^*CKO*^ mice than in *Smad3*^*-/-*^ mice, and the degree of elongation did not differ between *Smad2/3* double mutants and *Smad2*^*CKO*^ mice, suggesting that Smad2 has a more prominent role than Smad3 in elongation of the columnar zone (S4 Fig). Both *Smad2*^*CKO*^ and *Smad3*^*-/-*^ mice exhibited elongated hypertrophic zones compared to *Smad2*^*fx/fx*^ controls, and there was a significant elongation of the hypertrophic zone in *Smad2/3* double mutants compared to either *Smad2*^*CKO*^ or *Smad3*^*-/-*^ mice. Immunohistochemistry for PCNA was performed to test whether the increases in lengths of the colunmar zones were correlated with increased proliferation. A 2 to 3-fold increase in PCNA-positive cells was seen in the resting zones of *Smad2*^*CKO*^, *Smad3*^*-/-*^ and *Smad2/3* double mutant mice compared with control *Smad2*^*fx/fx*^ littermates; the degree of PCNA staining in the resting zone was similar in *Smad2/3* double mutants and *Smad2*^*CKO*^ mice (Fig 2C). No differences were detected in the columnar zone (Fig 2B and 2C). TUNNEL assays were performed to evaluate whether differences in cell survival contribute to the longer hypertrophic zones in mutants. No differences were detected (Fig 2D). These results indicate that loss of *Smad2* and/or *Smad3* promotes the entry of resting chondrocytes into the highly proliferative columnar phase, and that Smad2 appears to have a more prominent role than does Smad3. The increased lengths of the columnar and hypertrophic zones in mutants is consistent with an increased pool of chondrocytes transiting out of the resting zone and eventually undergoing hypertrophy. These findings suggest that Smad2 and Smad3 function to maintain the pool of resting chondrocytes in a quiescent state in neonatal growth plates.

### Increased *Ihh* RNA and protein levels in *Smad2*, *Smad3*, and *Smad2/3* mutant chondrocytes

**Fig 3:**
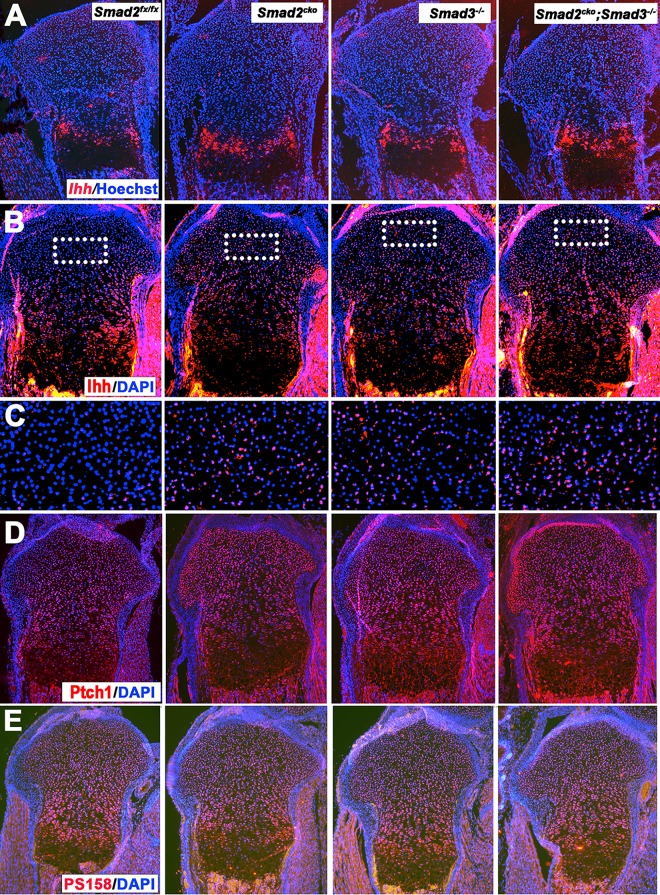
Increased *Ihh* mRNA and protein levels and activity in *Smad2* and *Smad3* mutant growth plates. All images are P0 proximal tibias **(A)** RNA *in situ* hybridization for *Ihh* showing increased expression in *Smad2*^*CKO*^, *Smad3*^*-/-*^, and double mutant growth plates. **(B)** Immunostaining for Ihh protein showing diffusion into the resting zones *of Smad2*^*CKO*^, *Smad3*^*-/-*^, and double mutant growth plates. **(C)** Magnifications of the boxed regions in (B). **(D)** Immunostaining for Ptch1, showing increased protein levels throughout the growth plate in mutants, particularly in the resting zone. **(E)** Immunostaining for phospho-Smad1/5/8 (PS158) protein showing no significant difference in levels of activated BMP signaling comparing *Smad2*^*CKO*^, *Smad3*^*-/-*^, or double mutant to control *Smad2*^*fx/fx*^ growth plates. *Smad2*^*CKO*^ = *Smad2*^*fx/fx*^*;Col2a1Cre*.

**Fig 4:**
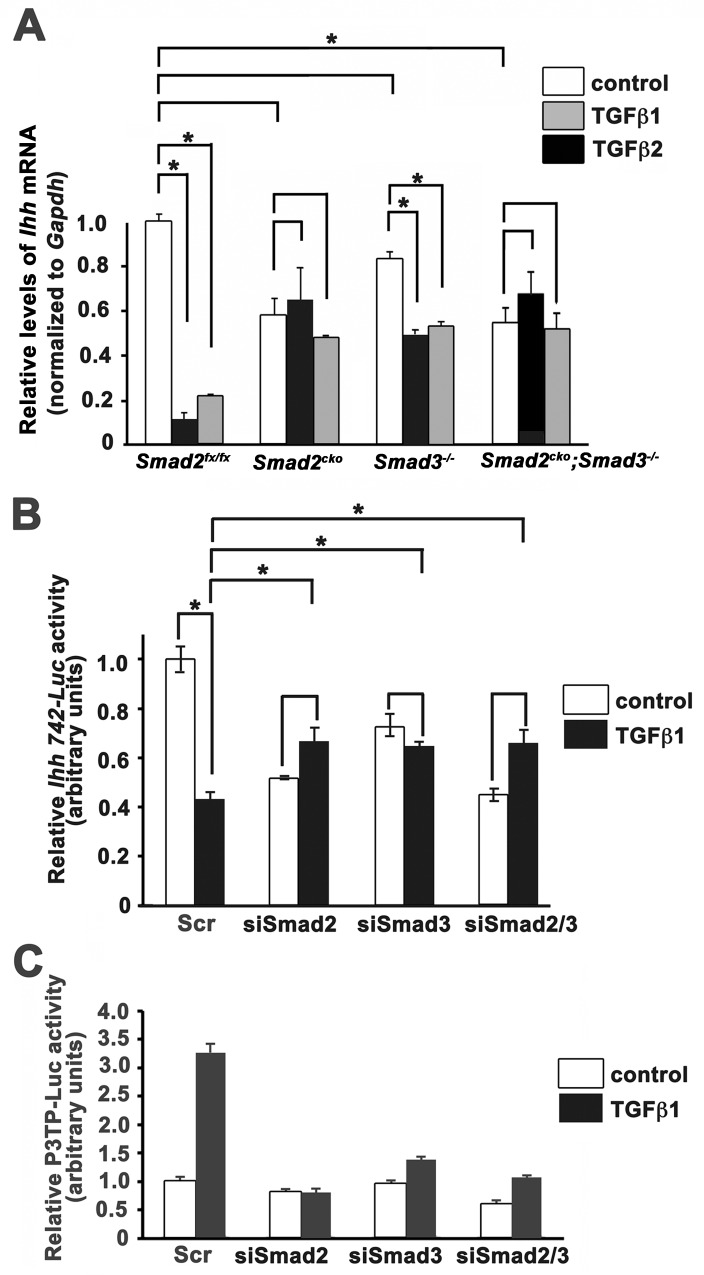
Smads 2 and 3 regulate *Ihh* levels in chondrocytes. **(A)** *Ihh* mRNA levels in primary rib chondrocytes isolated from P0 mice of the indicated genotypes. Chondrocytes were maintained in chondrogenic medium (see Material and Methods) to mature them to prehypertrophy. At this stage, they were treated with TGFβ1 (5 ng/ml) or TGFβ2 (5 ng/ml) for 24 hrs. *Ihh* RNA levels were assessed by quantitative real time PCR. Values were normalized using *Gapdh* and are plotted relative to control (*Smad2*^*fx/fx*^) + SE. **(B)** 742 bp *Ihh*-Luc activity in ATDC5 chondrocytes matured to prehypertrophy. Cells were treated with siRNAs against *Smad2* and/or *Smad3*, and then treated with TGFβ1 (5 ng/ml) for 24 hrs. Scr, scrambled siRNA control. **(C)** P3TP-Luc activity in ATDC5 chondrocytes under conditions of *Smad2/Smad3* knockdown, verifying regulation of this control reporter by Smad2 and Smad3. All experiments were performed in triplicate and repeated twice. Asterisks, p < 0.05. *Smad2*^*CKO*^ = *Smad2*^*fx/fx*^*;Col2a1Cre*.

*Ihh* is expressed in prehypertrophic chondrocytes and is a critical regulator of chondrocyte proliferation and differentiation. Since pSmads 2 and 3 are present in prehypertrophic chondrocytes (S1 Fig), RNA *in situ* hybridization was performed to assess *Ihh* RNA levels. The zone of *Ihh* expression is increased (Fig 3A), and qPCR quantification of RNA isolated from P0 growth plate cartilage showed that the level of *Ihh* RNA is increased in all three mutant strains compared with control *Smad2*^*fx/fx*^ littermates (S5 Fig), suggesting that Smad2 and Smad3 normally inhibit *Ihh* expression in the growth plate. *Smad2/3* double mutants had elevated levels of *Ihh* RNA and protein compared to both *Smad2*^*CKO*^ and *Smad3*^*-/-*^ mice, suggesting that both Smad2 and Smad3 contribute to elevated *Ihh* expression. As shown previously [34], Ihh protein was found in the control *Smad2*^*fx/fx*^ growth plate, with highest levels in the prehypertrophic and hypertrophic zones (Fig 3B). Ihh protein was detected in these regions in *Smad2* and *Smad3* single and double mutants, but unlike control *Smad2*^*fx/fx*^ littermates, was also detected in the resting zones (Figs 3B, 4C and S5). This suggests that the elevated *Ihh* RNA level in the prehypertrophic zone leads to increased diffusion of Ihh protein to the resting zone in mutants. *Patched1* (*Ptch1*) is a direct transcriptional target of Ihh signaling. Consistent with elevated levels of Ihh RNA and protein, immunostaining for Ptch1 demonstrated increased levels throughout the growth plates in mutants; this was most evident in the resting zones of *Smad2*^*CKO*^ and *Smad2/3* double mutant mice (Figs 3D and S5). *Ihh* is a downstream target of BMP signaling [35, 36], raising the possibility that the increased *Ihh* RNA expression is a consequence of increased BMP signaling in the growth plates of *Smad2*^*CKO*^, *Smad3*^*-/-*^ and *Smad2/3* double mutant mice. However, immunohistochemical examination in P0 growth plates revealed no obvious change of pSmad1/5/8 in mutant mice compared with control *Smad2*^*fx/fx*^ mice (Figs 3E and S5). In summary, the elevated level of *Ihh* RNA in the prehypertrophic zone, and increased domain of Ihh protein localization to the resting zone is correlated with an increase in the level and distribution of Ptch1. As Ihh promotes chondrocyte proliferation in the growth plate [37], the increased Ihh level and activity seen in *Smad2*^*CKO*^, *Smad3*^*-/-*^, and double mutants may contribute to the increased rate of proliferation in the resting zone in mutants.

### Direct effect of Smads 2 and 3 on *Ihh* promoter activity

The above findings indicate that Smads 2 and 3 act to decrease *Ihh* RNA levels in the neonatal growth plate. To investigate whether this effect is direct, primary rib chondrocytes were isolated, matured to the prehypertrophic phase by maintenance in chondrogenic differentiation medium, and then treated with TGFβ1 or TGFβ2. TGFβ1 and TGFβ2 decreased *Ihh* RNA levels in control *Smad2*^*fx/fx*^ chondrocytes (Fig 4A). However, the ability of TGFβ to inhibit *Ihh* expression was impaired in *Smad3*^*-/-*^ mutant chondrocytes, and abolished in *Smad2*^*CKO*^ and *Smad2/3* double mutant chondrocytes. However, a caveat of these findings is that although levels of *Ihh* RNA in primary chondrocytes from *Smad2*^*CKO*^ and *Smad3*^*-/*-^ mice were not reduced compared to control *Smad2*^*fx/fx*^ chondrocytes under basal conditions (no serum and no growth factor addition), levels of *Ihh* RNA were reduced in prehypertrophic chondrocytes from *Smad2/3* double mutants under basal conditions (Fig 4A). It is unclear whether this reflects a defect in the ability of *Smad2/3* double mutant primary chondrocytes to undergo timely differentiation *in vitro*. Alternatively, Smads2/3 may play a role in maintaining basal levels of *Ihh* RNA. Overall however, the *in vivo* (Fig 3A) and *in vitro* (Fig 4A) results indicate that Smad2 and Smad3 are required to inhibit *Ihh* expression in prehypertrophic chondrocytes in the neonatal growth plate.

To test whether Smads 2/3 play a direct role in regulating *Ihh* promoter activity, a luciferase reporter containing the proximal 742 bp of the mouse *Ihh* promoter [38] was transfected into ATDC5 chondrocytic cells. After culture in differentiation medium to induce prehypertrophy, the cells were treated with TGFβ1 for 24 hours. To test whether Smad2 and/or Smad3 mediate *Ihh* inhibition, Smad2 and/or Smad3 levels were knocked down by transfection of verified siRNAs. Reporter assays showed that TGFβ1 inhibits *Ihh* promoter activity in control chondrocytes by > 50% (Fig 4B). P3TP-Luc activity was used as a positive control for TGFβ activity, and showed robust activation under the same conditions (Fig 4C). Consistent with the analysis *in vivo* (Figs 3A and S5), knockdown of Smad2 and/or Smad3 blocked the inhibitory effect of TGFβ on *Ihh* promoter activity.

### Smad2 and Smad3 binding elements are required for inhibition of *Ihh* promoter by TGFβ

**Fig 5:**
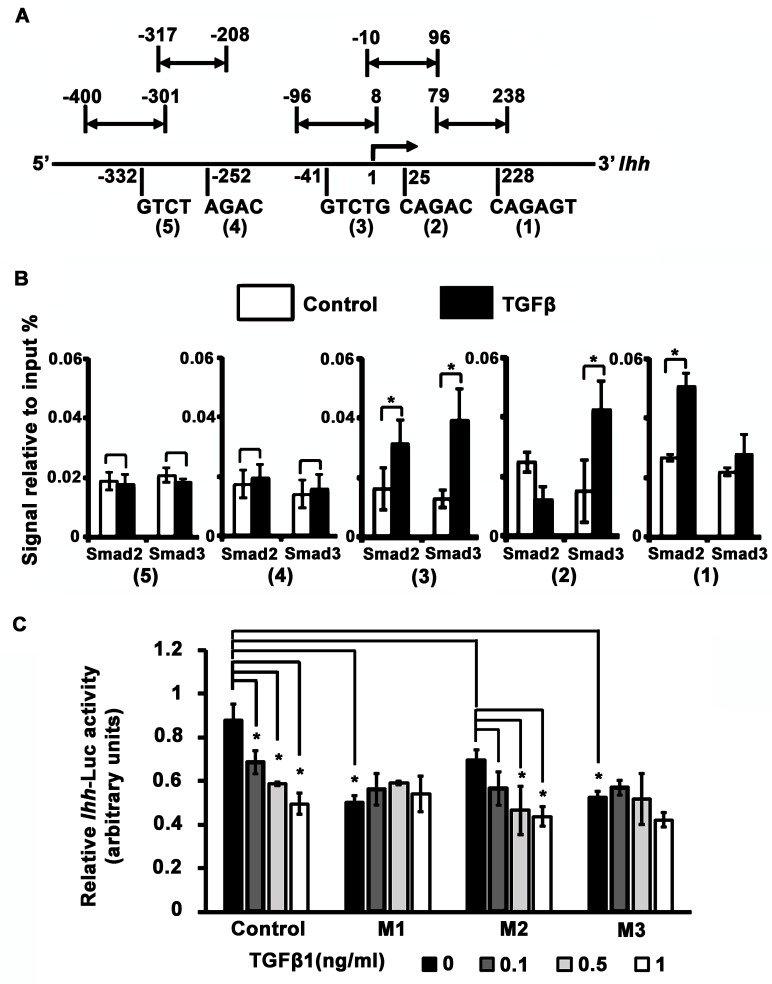
Smads 2 and 3 bind to distinct sites on the *Ihh* promoter and recruit different repressors. **(A)** Schematic diagram of the proximal mouse 5’ *Ihh* promoter and enhancer. The sequences and approximate locations of 5 putative canonical SBEs sites (1)–(5) are indicated below the promoter and enhancer. 1 is defined as the transcription start site. The primer locations used to analyze the SBEs are indicated above the promoter. See Materials and Methods for primer sequences. **(B)** ChIP analysis of Smad2 and Smad3 binding to each of the SBEs in the proximal *Ihh* promoter in ATDC5 cells matured to prehypertrophy and subsequently treated with TGFβ1 (5 ng/ml) for 24 hrs. **(C)** Mutated *Ihh*742-Luc activity with one of the SBEs missing. Luc constructs M1, M2 and M3 lose SBE1, SBE2 and SBE3 respectively by mutation in a *Ihh*742-Luc construct (control). Reporter assays were performed in ATDC5 chondrocytes treated with different concentration of TGFβ1 (0, 0.1, 0.5 and 1 ng/ml) for 24 hrs. Asterisk in (B) and (C), p < 0.05. All experiments were performed in triplicate and repeated twice.

Activated Smads 2 and 3 bind to Smad binding elements (SBEs) that contain (C)AGAC motifs [39–42]. ChIP-chip/ChIP-seq studies have confirmed that the SBE is enriched in Smad2/3 binding regions. [43–47]. *In silico* examination of the 742bp proximal *Ihh* promoter identified 5 putative SBEs, designated S1 to S5 (Fig 5A). ChIP analysis performed in ATDC5 cells for Smad2 and Smad3 binding showed differential occupation of S1-S3 in the presence of TGFβ; neither Smad2 nor Smad3 associated with S4 or S5 (Fig 5B). In addition, comparative Genomic Analysis using the UCSC Genome Browser (*http://genome.ucsc.edu*) [48] showed that S1, S2 and S3 are 100% conserved in the mouse, rat, human and dog genomes (S6 Fig). At S1, TGFβ increased binding of Smad2 but not Smad3 (Fig 5B). In contrast, at S2, TGFβ increased the association of Smad3, but had no effect on Smad2 binding. Association of both Smad2 and Smad3 is increased by TGFβ at S3. These results reveal 3 binding elements for pSmad2/3 within the 742 bp *Ihh* promoter region, and demonstrate that Smad2 and Smad3 have both common and distinct binding patterns.

To examine whether S1-S3 mediate the inhibitory effect of TGFβ on *Ihh*, six nucleotides covering the conserved SBE regions in S1, S2 and S3 were replaced with PsiI recognition sites in p*Ihh*742-Luc to generate mutated constructs M1, M2 and M3, respectively. Reporter assays revealed no significant inhibitory effect of TGFβ on activity of the M1 and M3 constructs, and the inhibitory effect of TGFβ on activity of the M2 construct was decreased compared with of the control construct (Fig 5C). These results indicated that S1 and S3 exert more inhibitory function than S2.

Similar to the results in Fig 4B showing lower basal activity of the *Ihh* promoter in *Smad2/3* double mutant primary chondrocytes, M1 and M3 exhibited decreased basal activity compared to the control *Ihh* promoter (Fig 5C), indicating that S1 and S3 also play a role in mediating basal activity of the *Ihh* promoter. The basis for the lower basal activity of the *Ihh* promoter is unclear. However, BMP signaling enhances *Ihh* promoter activity and association of Smad4 with SBEs in mouse teratocarcinoma P19 cells [35]. This raised the possibility that S1-S3 might recruit Smad4 to the *Ihh* promoter in response to BMPs, and that this recruitment is required for basal *Ihh* promoter activity. We therefore compared the activities of M1, M2 and M3 in response to BMP treatment in ATDC5 cells. We observed slightly lower basal levels of activity as in Fig 5C, but found no significant differences in BMP-mediated induction between the control and mutant promoters (S7 Fig). Together, our data indicate that S1 and S3 are important for Smad2 and Smad3-mediated inhibition of *Ihh* expression. We also find that S1 and S3 are important for maintaining basal levels of *Ihh* expression, but that this activity is not due to a role for these SBEs in mediating BMP responsiveness in chondrocytes.

### Smad2 and Smad3 associate with different transcriptional repressors on the *Ihh* promoter

**Fig 6:**
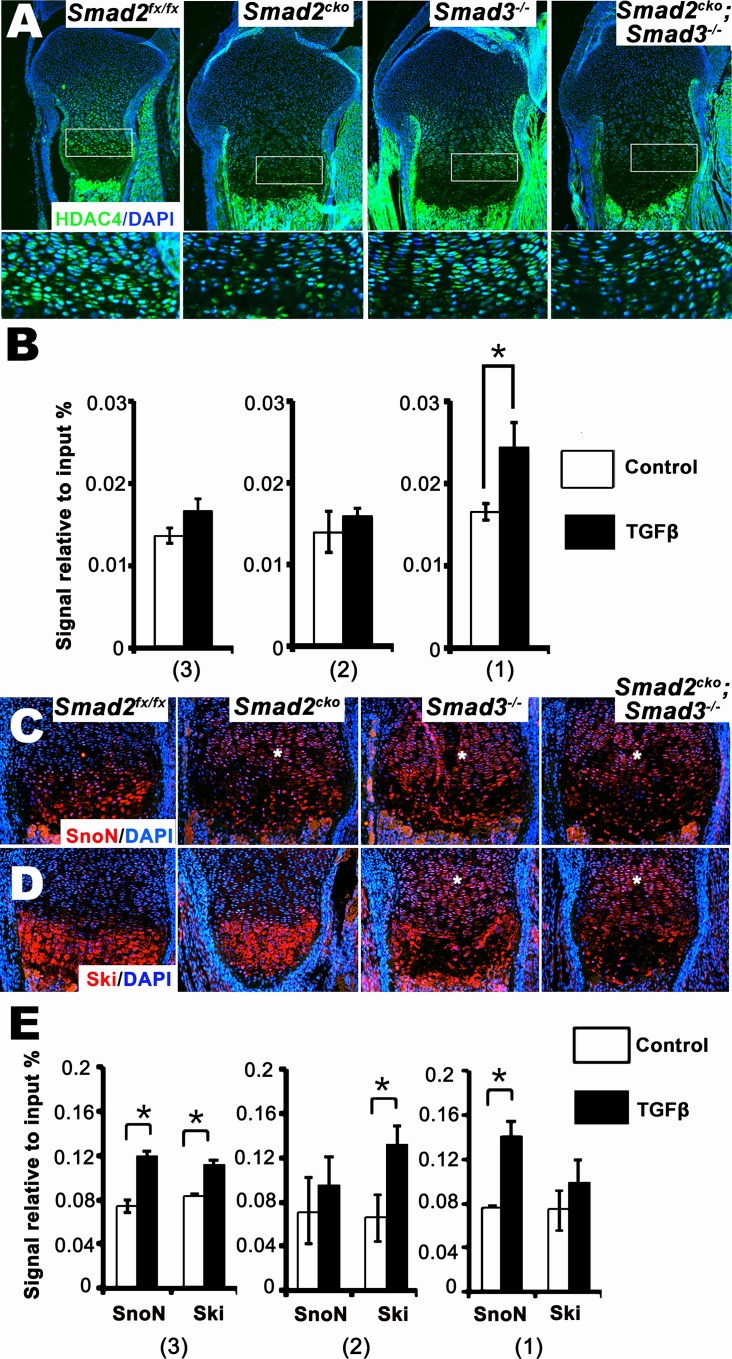
Hdac4, SnoN, and Ski protein distribution in the neonatal growth plate and binding to S1-3 in the *Ihh* promoter. **(A)** Immunohistochemical staining of Hdac4 in E18.5 proximal tibias. Bottom panel shows magnifications of the boxed regions in top panel, representing the lower columnar and prehypertrophic zones. **(B)** ChIP analysis of Hdac4 binding to SBE 1–3 in ATDC5 cells. **(C and D)** Immunohistochemical staining of SnoN and Ski in E18.5 proximal tibias. Asterisks in (C and D) indicated SnoN or Ski present in columnar zone cells. **(E)** ChIP analysis of SnoN and Ski binding to SBE 1–3 in ATDC5 cells. Cells in (B and E) were matured to prehypertrophy and subsequently treated with TGFβ1 (5 ng/ml) or non-treated (Control) for 24 hrs. Asterisk in (B and E), p = or < 0.05. All experiments were performed in triplicate and repeated twice. *Smad2*^*CKO*^ = *Smad2*^*fx/fx*^*;Col2a1Cre*.

Smad2 and Smad3 interact with a variety of DNA-binding proteins in different contexts. Hdac4 is expressed in prehypertrophic and hypertrophic zones of the growth plate and represses hypertrophy by binding to and blocking Runx2 activity [49]. Although Runx2 is a potent inducer of *Ihh* expression [50], whether or not Hdac4 directly regulates *Ihh* expression is unknown. Immunostaining confirmed that Hdac4 is present in the lower proliferative, prehypertrophic and hypertrophic zones of control *Smad2*^*fx/fx*^ P0 growth plates, and is localized in the nucleus, as reported previously [49] (Fig 6A). The level of Hdac4 protein was greatly diminished in the growth plates of *Smad2*^*CKO*^ and double mutant mice (Figs 6A and S8A). The percentage of cells expressing nuclear Hdac4 was significantly decreased at the border of the prehypertrophic and lower columnar zones of *Smad2/3* double mutants compared to either *Smad2*^*CKO*^ or *Smad3*^*-/-*^ mice (S8A Fig), indicating that both Smad2 and Smad3 contribute to decreased Hdac4 nuclear localization. To test whether the effect on Hdac4 expression is transcriptional, RNA levels were examined in growth plate cartilage from control *Smad2*^*fx/fx*^, *Smad2*^*CKO*^, *Smad3*^*-/-*^, and double mutant neonatal mice. No significant differences were observed (S8B Fig). These results suggest that both Smad2 and Smad3 regulate Hdac4 localization and/or stability at the border of the lower columnar and prehypertrophic zones, but Smad2 has a greater impact than Smad3.

Next, we tested whether Smad2 and/or Smad3 associate directly with Hdac4 in chondrocytes. Immunoprecipitation assays revealed weak association between Hdac4 and Smad2 without TGFβ, and this association was increased by TGFβ; no association of Hdac4 with Smad3 was detected (S8C Fig). Because this analysis revealed that Hdac4 associates with Smad2, we used ChIP to test whether Smad2 and Hdac4 interact on the *Ihh* promoter. TGFβ increased the level of Hdac4 associated with SBE1, the site exhibiting the highest level of pSmad2 binding; we did not detect Hdac4 binding to SBE3, in spite of the fact that Smad2 binds to this site (Fig 6B). These findings suggest that Smad2 regulates Hdac4 protein expression or stabilization, and also recruits Hdac4 to a repressive complex at SBE1 in the *Ihh* promoter.

SnoN is a repressor that can be induced by TGFβ [51, 52]. It is expressed in prehypertrophic chondrocytes, and inhibits chondrocyte hypertrophy [53]. Immunostaining of P0 growth plates showed that SnoN was expressed and localized within the nucleus in prehypertrophic and hypertrophic zones in control *Smad2*^*fx/fx*^ mice (Fig 6C). There was no obvious change in SnoN protein levels in prehypertrophic and hypertrophic chondrocytes in *Smad2*^*CKO*^, *Smad3*^*-/-*^ and double mutant mice (Fig 6C). However, there was an increase in SnoN protein levels in the proliferative zones in all mutant strains (Fig 6C). This increase in protein levels was not due to increased levels of *SnoN* RNA; in fact, *SnoN* RNA levels were decreased in *Smad3*^*-/-*^ and *Smad2/*3 mutant growth plates (S8D Fig). TGFβ signaling leads to rapid degradation of SnoN in a Smad2/Smad3-dependent manner [54]. Hence the elevated protein levels in mutants in spite of reduced mRNA levels may reflect increased SnoN stablity in the absence of Smads 2 and 3.

Ski proteins can suppress TGFβ and BMP signaling by binding to R-Smads and Smad4 [55, 56]. Ski was localized to the prehypertrophic and hypertrophic zones in *Smad2*^*fx/fx*^ mice (Fig 6D). There was no apparent change in protein levels in prehypertrophic and hypertrophic zones in any mutant strain, but there were increased levels of immunostaining in the proliferative zones in *Smad3*^*-/-*^ and *Smad2/3* double mutant strains. As is the case for SnoN, this effect appears to be posttranscriptional because loss of *Smad3* led to reduced *Ski* RNA levels in spite of the elevated protein levels (S8E Fig).

ChIP was performed to test whether SnoN and Ski might mediate the repressive effects of Smads 2 and 3 on *Ihh* expression in chondrocytes. This analysis showed that TGFβ increased the association of SnoN and Ski with SBE1, SBE2, and SBE3 in the *Ihh* promoter in ATDC5 cells (Fig 6E). SBE1 exhibited greater association with SnoN than with Ski, whereas more Ski than SnoN bound to SBE2; SnoN and Ski associated with SBE3 at similar levels (Fig 6E). These results parallel the differential binding of Smads 2 and 3 to these sites (Fig 5B), suggesting that SnoN and Ski may interact preferentially with Smad2 and Smad3, respectively. In addition, siRNA knockdown of *SnoN* abolished the ability of TGFβ to repress *Ihh*742-luc reporter activity; siRNA against *Ski* and *Hdac4* significantly reduced the ability of TGFβ to repress *Ihh*742-luc reporter activity (S9 Fig). Overall, the data suggest that both Smad2 and Smad3 mediate suppression of *Ihh* transcription by binding to distinct SBEs and associating with different repressors, but that Smad2 has a greater impact on *Ihh* RNA levels than Smad3.

## Discussion

Canonical TGFβ and activins transduce signals through Smad2 and Smad3 [57, 58]. Smad2 and Smad3 interact with different transcriptional regulators on DNA and can bind to distinct sites [32, 58]. Although Smad2 and Smad3 have similar functions in a number of contexts [32, 59, 60], they exert distinct, and even opposing, effects in others [32, 61, 62]. Whether this is the case in cartilage was unknown. Analysis of *Smad3*^*-/-*^ mice demonstrated previously that Smad3 suppresses chondrocyte hypertrophy [12]. The finding that overexpression of Smad2 can block the accelerated chondrocyte maturation seen in *Smad3*^*-/-*^ chondrocytes suggested that Smad2 and Smad3 exert at least some similar functions *in vitro* [33]. A limitation of the previous study as well as ours is the use of global *Smad3* mutants, raising the possibility that some aspects of the *Smad3* mutant growth plate phenotype are due to effects on other cell types, such as the perichondrium. However, direct effects on chondrocytes seem plausible based on *in vitro* studies [63, 64] and our results, which document direct effects in *Smad3*-deficient neonatal chondrocytes. Nonetheless, loss of *Smad2* appears to have a greater impact in the non-hypertrophic zone chondrocytes of the growth plate during embryogenesis than does loss of *Smad3*.

We found that both Smad2 and Smad3 are essential for chondrogenesis *in vivo* to inhibit proliferation and hypertrophy. Some of the alterations in proliferation, maturation, and gene expression were more pronounced in *Smad2*^*CKO*^*;Smad3*^*-/-*^ double mutants than in single mutants, indicating that Smads 2 and 3 exert similar functions in the growth plate. This may be mediated in part by the ability of Smads 2 and 3 to repress *Ihh* expression. Elevated *Ihh* RNA levels in mutants were correlated with increased levels of Ihh protein and its direct target Ptch1 in the resting zone. Although altered matrix properties in *Smad2/3* mutants may contribute to increased Ihh protein diffusion, it is likely that the elevated *Ihh* mRNA levels seen in these mutants are also responsible for the elevated Ihh protein levels in the resting zone and elevated Ptch1 levels throughout the growth plate. In accordance, the growth plates of mutants are lengthened at midgestation stages and P0. This is consistent with previous studies of *Ihh* function in the growth plate, where depletion of *Ihh* in prenatal cartilage caused a loss of columnar structure and dwarfism [65]. In spite of the longer growth plates at these stages, double mutants exhibit postnatal dwarfism. This can be explained if the accelerated rate of entry of resting chondrocytes into the proliferative columnar phase leads to premature depletion of the pool of resting chondrocytes and an inability to sustain growth plate elongation at postnatal stages. The precise role of Ihh in the postnatal dwarfism in *Smad2/3* double mutants is unclear; postnatal loss of *Ihh* in cartilage leads to dwarfism [66]. On the other hand, Ihh can promote terminal hypertrophic maturation, which could lead to dwarfism, in cells outside the range of PTHrP [67, 68]. Additional studies at postnatal stages would be needed to identify the mechanism underlying the postnatal dwarfism phenotype in *Smad2/3* mutants. We speculate that direct effects of Smad2 and Smad3 on genes regulating cell cycle progression may contribute.

Interestingly, the growth plate phenotype at the neonatal stage in *Smad2/3* double mutants is distinct from that seen in mice lacking the type II TGFβ receptor TβRII (*Tgfbr2*) in cartilage [21]. TβRII is required for responsiveness to all TGFβs. *Tgfbr2*^*CKO*^ mice, which were generated using the same *Col2a1-Cre* allele used here, exhibit defects in formation of intervertebral discs (IVD), but no apparent alterations in chondrocyte differentiation in axial or appendicular elements [21]. Obvious defects in IVD formation were not observed at birth in *Smad2/3* double mutants, but there were clear defects in the tibial growth plates. There are several possible explanations for these differences. Loss of *Tgfbr2* impacts both canonical Smad2/3 and non-canonical pathways. Hence, the axial defects seen in neonatal *Tgfbr2*^*CKO*^ mice but not in neonatal *Smad2/3* double mutants could reflect the actions of non-canonical TGFβ pathways. Furthermore, the growth plate defects at neonatal stages seen in *Smad2/3* double mutants but not in *Tgfbr2*^*CKO*^ mice could reflect a role for Smads 2 and 3 in signaling mediated by ligands other than TGFβs, such as activins [69].

Our analysis suggests that Smads 2 and 3 may regulate *Ihh* RNA levels by binding to distinct elements in the *Ihh* promoter. Candidate SBEs can be predicted in the promoter regions of many genes, but these motifs are common, and the majority of them are not occupied by R-Smads when examined using ChIP-chip/ChIP-seq [70]. We found three SBEs within the proximal *Ihh* promoter that bind Smads 2 and 3. These sites exhibit differential recruitment of Smads 2 and 3, and differential association with distinct co-repressors. S1 and S3 in the *Ihh* promoter mediate more of the repressive activity of TGFβ on *Ihh* expression than does S2. A caveat of this study is that the *Ihh* promoter sequences that regulate *Ihh* expression in the growth plate have not yet been identified; *in vivo* mutagenesis studies will be required to identify these. However, the proximal promoter and enhancer region we investigated is the most highly conserved region among 60 mammalian species (S6 Fig), and this region was shown previously to mediate Smad4 effects on *Ihh* expression [35] as well as the impact of multiple transcription factors on *Ihh* expression in chondrocytes [38, 39, 50].

Our studies revealed that Smad2 and Smad3 associate with transcriptional inhibitors Hdac4, SnoN and Ski after TGFβ stimulation. Hdac4 inhibits Runx2 activity and *Ihh* expression [49]; SnoN and Ski repress the transcriptional activation activities of Smad2 and Smad3 [51, 52]; SnoN and Ski also inhibit Smad1/5/8 mediated transcription activity in ATDC5 cells and W-20-17 osteoblasts [53, 55]. The studies indicate that Smads 2 and 3 act to repress *Ihh* promoter activity *in vitro*, consistent with the elevated *Ihh* RNA and protein levels seen in *Smad2*^*CKO*^, *Smad3*^*-/-*^ and *Smad2/3* double mutant mice *in vivo;* however, they also showed that although chondrocytes isolated from *Smad2/3* double mutant chondrocytes are impaired in their ability to decrease *Ihh* RNA levels in response to TGFβ, they also exhibit decreased basal levels of *Ihh* mRNA compared with control cells (Fig 4A). The reason for lower basal *Ihh* RNA levels in *Smad2/3* chondrocytes *in vitro* is unclear. Combined loss of Smad2 and Smad3 may affect the ability of these cells to undergo differentiation *in vitro*. It is important to bear in mind that the isolated chondrocytes were maintained in the absence of added growth factors, a condition that does not mimic the intact growth plate, in order to study the direct role of TGFβ. Additional studies will be required to establish the role of Smad2 and Smad3 in basal *Ihh* expression. A low level of association of Smad2 and Smad3 with S1 and S3 may be required to maintain basal activity of the *Ihh* promoter. Evidence for this comes from the finding that basal levels of *Ihh* promoter activity are lower in M1 and M3 *Ihh* promoter constructs. However, it is also possible that these mutations have an impact on the binding of other factors that are required for basal promoter activity. Nonetheless, comparison of the effects of TGFβ vs. no growth factor reveals the importance of S1 and S3 for the inhibitory effects of TGFβ.

In summary, our studies reveal a role for Smad2 in the neonatal growth plate, and indicate that both Smad2 and Smad3 maintain the pool of resting chondrocytes in the growth plate. Future studies are warranted to investigate the function of Smad2 directly in postnatal articular cartilage.

## Materials and Methods

### Generation of *Smad2*^*fl/fl*^*;Col2-cre* and *Smad2*^*fl/fl*^*;Smad3*^*-/-*^*;Col2-cre* mice

*Smad2*^*fx/fx*^ mice [31] were intercrossed with the *Col2a1-Cre* deleter strain [71] to generate *Smad2*^*fx/fx*^*;Col2a1Cre* (*Smad2*^*CKO*^) mice. These mice were intercrossed with *Smad3*^*-/-*^ mice [27] to generate *Smad2*^*fx/fx*^*;Smad3*^*-/-*^*;Col2a1-Cre* (*Smad2/3* double mutant) mice. Primers for PCR genotyping include sense 5′-TGCTCTGTCCGTTTGCCG -3′ and anti-sense 5′-ACTGTGTCCAGACCAGGC-3′ for detecting the Col2-Cre allele, sense 5′-CCCGGTAAATCTACCCTAG-3′ and anti-sense 5′-TTTCAAAACTATATTTGCCCAAG-3′ for detecting the *Smad2*-floxed allele, sense 5′-GGATGGTCGGCTGCAGGTGTCC-3′ and anti-sense 5′-TGTTGAAGGCAAACTCACAGAGC-3′ for detecting the *Smad3* WT allele, sense 5′-GTTGCAGTGCACGGCAGATACACTTGCTGA-3′and anti-sense 5′-GCCACTGGTGTGGGCCATAATTCAATTCGC-3’ for detecting the *Smad3* mutant allele. Embryos and mice were on a mixed C57BL/6J/CD1 background and were maintained in accordance with the NIH Guide for the Care and Use of Laboratory Animals and were handled according to protocols approved by the institution’s subcommittee on animal care (IACUC).

### Ethics statement

This research was approved by the UCLA Animal Research Committee (ARC) under protocol 1995-018-71.

### Skeletal preparation and histology

Whole mount skeletal preparations were performed as described [72]. For histological analyses, paraffin sections were produced from E16.5, P (postnatal day) 0, 2 weeks, 1 month, and 4 month-old mice. Limb tissues were dissected and fixed in 4% paraformaldehyde in PBS. They were then decalcified with Immunocal (Decal Chemical Corp., Tallman, NY, USA) for 3 days at 4°C, embedded in paraffin, and cut at a thickness of 7 μm. Sections were stained with alcian blue (Sigma-Aldrich, A5268) and nuclear fast red (Sigma-Aldrich, N8002). Safranin-O staining was performed as described (Rosenberg, 1971). Heights of proliferative and hypertrophic zones were measured directly from images (n = 5) taken from each of five mice per genotype and significance was evaluated using Student’s t-test.

### Immunohistochemistry (IHC), RNA *in situ* hybridization and TUNEL assay

Paraffin sections were deparaffinized and rehydrated by passage through xylene and 100, 95, and 70% ethanol. Endogenous peroxidase activity was quenched by incubation for 15 min in 3% hydrogen peroxide. Samples were treated with 1mg/ml hyaluronidase for 30 min at 37° C. Sections were blocked with 5% goat serum, in TBS for 1 h at room temperature and incubated with 1:100 diluted primary antibody at 4° C overnight. Antibodies used were: phospho-Smad2 (Cell Signaling, Beverly, MA, USA, #3108), PCNA (Cell Signaling, #13110), Ihh (Abcam, ab52919), Patched1 (Novus Biologicals, NB200-118), Collagen X (Abcam, ab140230), SnoN (Santa Cruz, sc-9141), Ski (Santa Cruz, sc-9140) and Hdac4 (Cell Signaling, cs-2072). For colorimetric detection, sections were treated with secondary antibodies conjugated to HRP as per manufacturer’s instructions, and HRP visualized with EnzMet-TM HRP Detection Kit (Nanoprobes, Yaphank NY, #6001). Nuclei were counter-stained with fast red. For fluorescence detection, sections were treated with secondary antibody (1:500) labeled with fluorescent dyes (Cell Signaling, Goat anti-Rabbit Red:R37117, Goat anti-Rabbit Green: R37116, Goat anti-Mouse Red: A11032) at room temperature for 1 h, and nuclei were counter-stained with DAPI. Alternate sections used for IHC analysis were used for RNA *in situ* hybridization with 35^S^-labeled probes for *Ihh* [38, 65]. Apoptotic cells were detected by *in situ* terminal deoxynucleotidyltransferase deoxyuridine triphosphate nick end labeling (TUNEL) assay using the In Situ Cell Death Detection Kit (Sigma, #11684795910) following the manufacturer's instructions. All experiments were repeated on sections from at least three embryos of each genotype. All comparisons were between littermates.

### RNA isolation and quantitative PCR

Primary rib chondrocytes isolated from P0 mice were maintained in αMEM (Gibco, #12571) plus 10% FBS for 3 days in order to mature the cells to the prehypertrophic stage. After 4 hours of cell starvation, recombinant TGFβ1 or TGFβ2 (R&D Systems, Minneapolis, MN, USA) was added to the chondrocyte cultures at a concentration of 5 ng/ml followed by incubation in αMEM without FBS for 24 hours. Cells were then fixed in TRIzol. Total RNA was isolated by the phenol-chloroform method and converted to cDNA. The cDNA was amplified and quantified using SYBR Green reagent (Sigma) in a Stratagen-TM Mx3005P qPCR System (Thermo Scientific, USA). Primer sequences were *Ihh*: sense 5’-GACTCATTGCCTCCCAGAACTG-3’ and antisense 5’-CCAGGTAGTAGGGTCACATTGC-3’, *Gapdh* sense 5’-ACCAGGTGGTCTCCTCTGACTTCAA-3’ and antisense 5’-TACTCCTTGGAGGCCATGTGGG -3’.

### siRNA, DNA transfection and reporter assay

ATDC5 cells were plated at 1.5 × 10^5^ cells/well in 24-well plates. After 18 h, the cells were transfected with Lipofectamine. For siRNA transfection, 30 μM siRNA was added per well. Silencer Select Smad2 (s69492) and Smad3 (s69494) siRNAs and non-targeting siRNA (1193893) were from Thermo Fisher Scientific (Life Sciences, USA). For DNA transfection, 0.25 μg of pIhh742-Luc [38] (Addgene), P3TP-Luc (Addgene), and 0.025 μg of *Renilla* plasmids (Addgene) were added per well. 24 h post-transfection, medium was replaced with chondrogenic differentiation medium: α-MEM containing 5%FBS, 200 μg/ml ascorbic acid, 60 nm Na_2_SeO_3_, 10 μg/ml transferrin, 1% antibiotic. After 4-days of culture, cells were treated with TGFβ1 at a concentration of 5 ng/ml. Luciferase assays were performed 24 h later. Dual-luciferase reporter assay was performed using the Promaga kit (E1910) in a FLUOstar Omega system (BMG Labtech, Ortenberg, Germany) following the manufacturer's instructions. Data are presented as ratios of Luc/Renilla activity from at least three different experiments and each experiment was performed in triplicate for each DNA sample. The sequence information for the 742bp Ihh construct is available in Addgene. It includes the 264bp 5’ untranslated region and 478bp of proximal promoter sequence.

### Chromatin immunoprecipitation (ChIP)

ChIP for Smad2 and Smad3 was performed in ATDC5 cells that were matured to prehypertrophy by 4-days of culture in chondrogenic differentiation medium followed by treatment with or without TGFβ1 (5ng/ml) for 4 hours. ChIP was performed using a ChIP kit (Cell Signaling, #9005S) according to the manufacturer's instructions. In brief, after cross-linking and cell lysis, chromatin was sheared by sonication to yield DNA fragments in the range of 150 to 900 bp. 90% of the DNA fragments were in the range of 150–200 bp, confirmed by gel electrophoresis. 2% of the diluted cell supernatant was kept as input material to quantify DNA content of the samples. The supernatants were immunoprecipitated overnight at 4°C with antibodies against Smad2 (Cell Signaling, cs-5339s), Smad3 (Cell Signaling, cs-9523s), SnoN (Santa Cruz, sc-9141), Ski (Santa Cruz, sc-9140) or Hdac4 (Cell Signaling, cs-2072). For a negative control a rabbit IgG immunoprecipitation was performed in parallel using the same concentration as the ChIP antibody. DNA was isolated using phenol-chloroform followed by quantitative PCR analysis. ChIP-enriched DNA was quantitated using the Stratagen-TM Mx3005P qPCR System (Thermo Scientific, USA) with SYBR green PCR master mix (Sigma), using the absolute quantification method, in which ChIP DNA PCRs were run alongside a standard curve of genomic DNA. PCR signals were quantitated by normalization to the total input DNA reaction and the internal intergenic control primer pair (QIAGEN, #GPM100001C(-)01A). At least three independent samples were analyzed. Primer-amplified fragments between 100 and 200 bp were centered on the Smad2/Smad3 consensus binding sites. Primer sequences are as follows: SBE1, 5’-CTAACCGCGGGTCCCTTC-3’, and 5’-GCCTCGACTCTGAGCTGC-3’; SBE2, 5’-CATTTCCCCTCTCACTCGAC-3’, and 5’-GAAGGGACCCGCGGTTAG-3’; SBE3, 5’-CTTGCTGCAGGTTCGCTG-3’, and 5’-CGAGTGAGAGGGGAAATGGA-3’; SBE4, 5’-GGCATCTCCTGTCCAGGA-3’, and 5’-CTGCCTGCGATTGTCCTC-3’; SBE5, 5’-ACACCGTAGGCGGTTGTG-3’ and 5’-TCCTGGACAGGAGATGCC- 3’.

### Mutagenesis

Six nucleotides within SBE1, SBE2 or SBE3 in a luciferase construct pIhh742-Luc [38] were replaced by restriction endonuclease PsiI recognition sites (TTATAA) to generate three mutant constructs M1-Luc, M2-Luc, M3-Luc, respectively, using the QuikChange Site-Directed Mutagenesis Kit (Agilent Technologies, #200519). The primers for mutagenesis are as follows: M1-Luc, 5’-GCTTTATAACGAGGCGCCGAGGGGGA- 3’ and 5’-TCGTTATAAAGCTGCCCGGCTCGCCG—3’; M2-Luc, 5’-CCGTTATAAGCAGCAGCTCCCGCTCT- 3’ and 5’-TGCTTATAACGGCGCAGCCCGGGGTC—3’; M3-Luc, 5’-TGCTTATAACGCGGGTCCCGAGCCCG—3’ and 5’-GCGTTATAAGCACCCTATCCATGTCC—3’.

### Co-Immunoprecipitation (Co-IP) and western blot

Co-IP was performed in ATDC5 cells that were matured to prehypertrophy and treated with TGFβ1 (5ng/ml) or not, as described above. The Pierce-TM Co-Immunoprecipitation Kit (ThermoFisher, #26149) was used according to the manufacturer's instructions. Protein concentrations were determined using the Coomassie Plus Protein Assay kit (Pierce, Rockford, IL, USA). SDS-PAGE was used to separate the protein extracts (30 μg). After transfer to a polyvinylidene fluoride (PVDF) membrane (NEN Life Science Products, Boston, MA, USA), and blocking with 5% milk, the blots were probed with rabbit anti-Hdac4 (1 μg/ml). After washing, the membrane was incubated with appropriate HRP–conjugated secondary antibody (Sigma) for 2 h at room temperature. The immune complexes were detected using ECL substrate (Pierce, Rockford, IL, USA). The blot was repeated using two independent cell preparations.

### Statistical analysis

Statistical comparisons were made between the groups using either ANOVA or Student’s t-test as appropriate. P values of <0.05 were considered significant and are denoted in each of the figures.

## Supporting Information

S1 Fig Localization of pSmad2/3 in growth plate cartilage. (A-D) Immunohistochemical staining of E16.5 proximal tibias. (E-H) Immunofluorescent staining of P0 proximal tibias. Approximate locations of the resting, proliferative, prehypertrophic, and hypertrophic zones are indicated by brackets. *Smad2*^*CKO*^ = *Col2a1-Cre;Smad2*^*fx/fx*^.
(TIF)

S2 Fig Subtle skeletal defects in P0 *Smad2* and *Smad3* mutants. (A) Lateral views of P0 littermates demonstrating reduced crown-rump length in *Smad2/3* (*Smad2*^*CKO*^*;Smad3*^*-/-*^*)* double mutants compared with *Smad2*^*fx/fx*^ littermates (90.3%, p < 0.05; n = 4). (B) Ventral views of skulls. Arrowhead, delayed ossification of the occipital condyle in double mutants. (C) Frontal (ventral) views of ribcages. Arrow highlights the shortened sternum and birfurcated xiphoid process (xp) in double mutants. (D) Ventral views of lumbar vertebrae, showing delayed ossification of the centra in double mutants (arrow). (E) C1 (atlas) and C2 (axis). Arrows show delayed ossification of the centra in *Smad2*^*CKO*^ and *Smad2/3* (*Smad2*^*CKO*^*;Smad3*^*-/-*^*)* double mutants. (F) Measurements of P0 tibial lengths (n = 5 per genotype) showing no significant differences. *Smad2*^*CKO*^ *=* (*Smad2*^*fx/fx*^*;Col2a1Cre*), *S2*^*fx/fx*^ = *Smad2*^*fx/fx*^, *S2*^*CKO*^ = (*Smad2*^*fx/fx*^*;Col2a1Cre*), *S3*^*-/-*^ = *Smad3*^*-/-*^, *S2*^*CKO*^*;S3*^*-/-*^ = (*Smad2*^*fx/fx*^*;Col2a1Cre;Smad3*^*-/-*^).
(TIF)

S3 Fig Postnatal dwarfism in *Smad2*^*CKO*^*;Smad3*^-/-^ mice. **(A)** Cleared skeletal preparations of P7 mice showing decreased axial length in *Smad2/3* (*Smad2*^*CKO*^*;Smad3*^*-/-*^*)* double mutants. **(B and C)** P14 and P30 littermates, showing that *Smad2*^*CKO*^ and *Smad3*^*-/-*^ mice are not obviously smaller than control *Smad2*^*fx/fx*^ littermates, but *Smad2/3* double mutants are smaller (N = 3, P<0.05). *Smad2*^*CKO*^ = *Smad2*^*fx/fx*^*;Col2a1Cre*.
(TIF)

S4 Fig Lengths of resting zones, columnar zones and hypertrophic zones in growth plates. Lengths of zones were measured directly from images (n = 5) through P0 proximal tibial growth plates of littermate mice from each of 5 mice per genotype. RZ = resting chondrocyte zone, CZ = columnar chondrocyte zone, and HZ = hypertrophic chondrocyte zone. Measurements were performed by individuals blinded to genotype. Significance was established using Student's *t*-test. *S2*^*fx/fx*^ = *Smad2*^*fx/fx*^, *S2*^*CKO*^ = (*Smad2*^*fx/fx*^*;Col2a1Cre*), *S3*^*-/-*^ = *Smad3*^*-/-*^, *S2*^*CKO*^*;S3*^*-/-*^ = (*Smad2*^*fx/fx*^*;Col2a1Cre;Smad3*^*-/-*^).
(TIF)

S5 Fig Increased *Ihh* RNA and protein levels in *Smad2* and *Smad3* mutant growth plate cartilage. Levels of *Ihh* RNA were determined by quantitative real time PCR on RNA isolated from P0 growth plate cartilage. Values were normalized using *Gapdh* and are plotted relative to control (*Smad2*^*fx/fx*^) + SE (n = 3). Levels of Ihh, Ptch1 and pSmad1/5/8 proteins in resting chondrocyte zone were determinded by counting the percentage of immunohistochemistry (IHC) staining positive cells over total cells (n = 3). Significance was established using Student's *t*-test. *S2*^*fx/fx*^ = *Smad2*^*fx/fx*^, *S2*^*CKO*^ = (*Smad2*^*fx/fx*^*;Col2a1Cre*), *S3*^*-/-*^ = *Smad3*^*-/-*^, *S2*^*CKO*^*;S3*^*-/-*^ = (*Smad2*^*fx/fx*^*;Col2a1Cre;Smad3*^*-/-*^).
(TIF)

S6 Fig Conservation of the SBEs in *Ihh* promoter. Comparative Genomic Analysis in the 5’ proximal *Ihh* promoter and transcription enhancer region using UCSC Genome Browser (*http://genome.ucsc.edu*) showed that Smad Binding Elements SBE1(CAGAGT), SBE2(CAGAC) and SBE3(GTCTG) are 100% conserved in mouse, rat, human and dog genomes.
(TIF)

S7 Fig No difference in BMP induction between control and mutant *Ihh* promoters. ATDC5 chondrocytes were transfected with control or mutant *Ihh*-promoter constructs and then treated with BMP2 (200 ng/ml) for 4 hrs. BMP2 significantly increased luciferase activities of all control and mutant constructs. There is no significant difference of BMP induction between control and mutant constructs. All experiments were performed in triplicate and repeated twice. Asterisks, p < 0.05.
(TIFF)

S8 Fig Smad2 and Smad3 regulate RNA and protein levels of Hdac4, SnoN and Ski in growth plate cartilage. (A) Levels of Hdac4 proteins in the nucleus of prehypertrophic chondrocyte were determinded by counting the percentage of immunohistochemistry (IHC) staining-positive cells with nuclear signal over total cell numbers in the prehypertrophic zone (n = 3). (B) Quantitative real time PCR showed *Hdac4* RNA level in growth plate cartilage (n = 3). (C) Co-IP analysis of Hdac4 associating with Smad2 and Smad3 in ATDC5 cells. The western blot images of Hdac4 associated with Smad2 were quantified using Adobe Photoshop CS3. The value for TGFβ1 treatment is expressed relative to control. (D-E) Quantitative real time PCR showed *SnoN* and *Ski* RNA level in growth plate cartilages. Values in B, D-E were normalized using *Gapdh* and are plotted relative to control (*Smad2*^*fx/fx*^) + SE (n = 3). Significance was established using Student's *t*-test. Asterisk in A, D-E, p < 0.05. *S2*^*fx/fx*^ = *Smad2*^*fx/fx*^, *S2*^*CKO*^ = (*Smad2*^*fx/fx*^*;Col2a1Cre*), *S3*^*-/-*^ = *Smad3*^*-/-*^, *S2*^*CKO*^*;S3*^*-/-*^ = (*Smad2*^*fx/fx*^*;Col2a1Cre;Smad3*^*-/-*^).
(TIF)

S9 Fig SnoN, Ski, Hdac4 are required for the inhibitiory effect of TGFβ on *Ihh* promoter activity. 742 bp *Ihh*-Luc activity in ATDC5 chondrocytes matured to prehypertrophy. Cells were treated with siRNAs against SnoN, Ski and Hdac4 expression, and then treated with TGFβ1 (2 ng/ml) for 24 hrs. Scr, scrambled siRNA control. All experiments were performed in triplicate and repeated twice. Significance was established using Student's *t*-test.
(TIFF)
