# Supplementary figures and images for: Smad2 and Smad3 Regulate Chondrocyte Proliferation and Differentiation in the Growth Plate
Source: PLoS Genet. 2016 Oct 14;12(10):e1006352. doi: 10.1371/journal.pgen.1006352 (PMC5065210; doi:10.1371/journal.pgen.1006352)

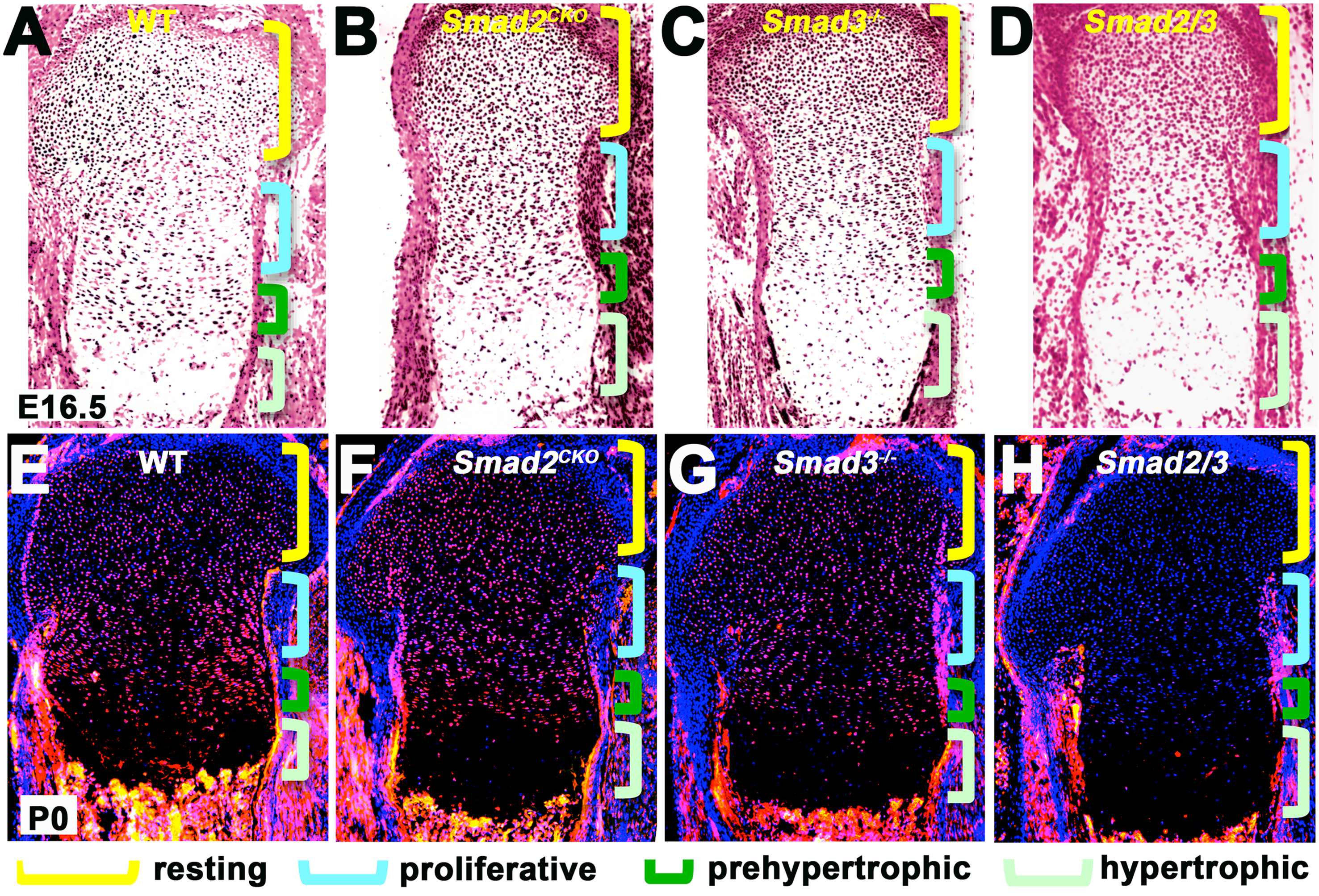

Supplement: S1 Fig — (A-D) Immunohistochemical staining of E16.5 proximal tibias. (E-H) Immunofluorescent staining of P0 proximal tibias. Approximate locations of the resting, proliferative, prehypertrophic, and hypertrophic zones are indicated by brackets. Smad2CKO = Col2a1-Cre;Smad2fx/fx. (TIF) [file pgen.1006352.s001.tif]

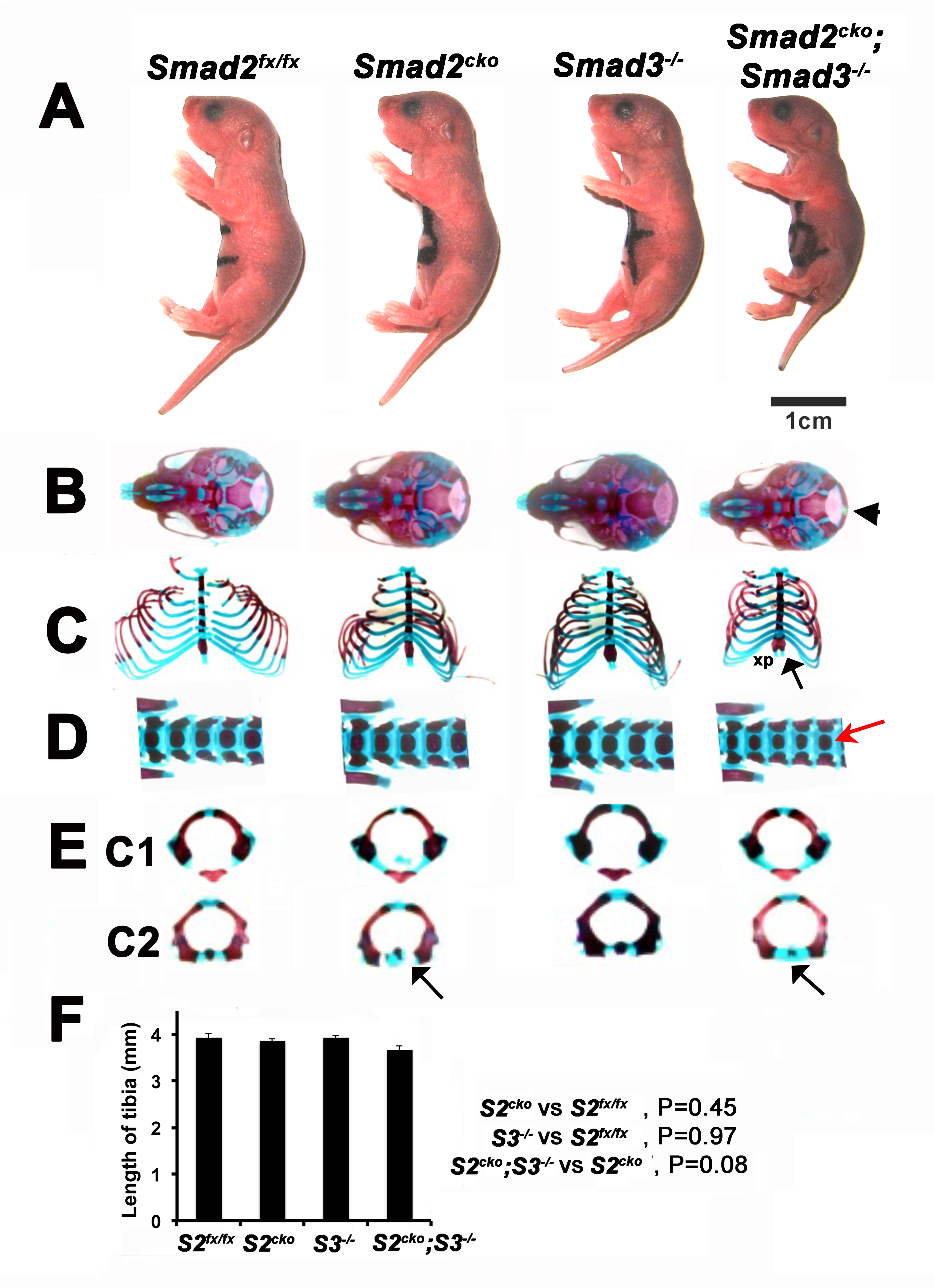

Supplement: S2 Fig — (A) Lateral views of P0 littermates demonstrating reduced crown-rump length in Smad2/3 (Smad2CKO;Smad3-/-) double mutants compared with Smad2fx/fx littermates (90.3%, p < 0.05; n = 4). (B) Ventral views of skulls. Arrowhead, delayed ossification of the occipital condyle in double mutants. (C) Frontal (ventral) views of ribcages. Arrow highlights the shortened sternum and birfurcated xiphoid process (xp) in double mutants. (D) Ventral views of lumbar vertebrae, showing delayed ossification of the centra in double mutants (arrow). (E) C1 (atlas) and C2 (axis). Arrows show delayed ossification of the centra in Smad2CKO and Smad2/3 (Smad2CKO;Smad3-/-) double mutants. (F) Measurements of P0 tibial lengths (n = 5 per genotype) showing no significant differences. Smad2CKO = (Smad2fx/fx;Col2a1Cre), S2fx/fx = Smad2fx/fx, S2CKO = (Smad2fx/fx;Col2a1Cre), S3-/- = Smad3-/-, S2CKO;S3-/- = (Smad2fx/fx;Col2a1Cre;Smad3-/-). (TIF) [file pgen.1006352.s002.tif]

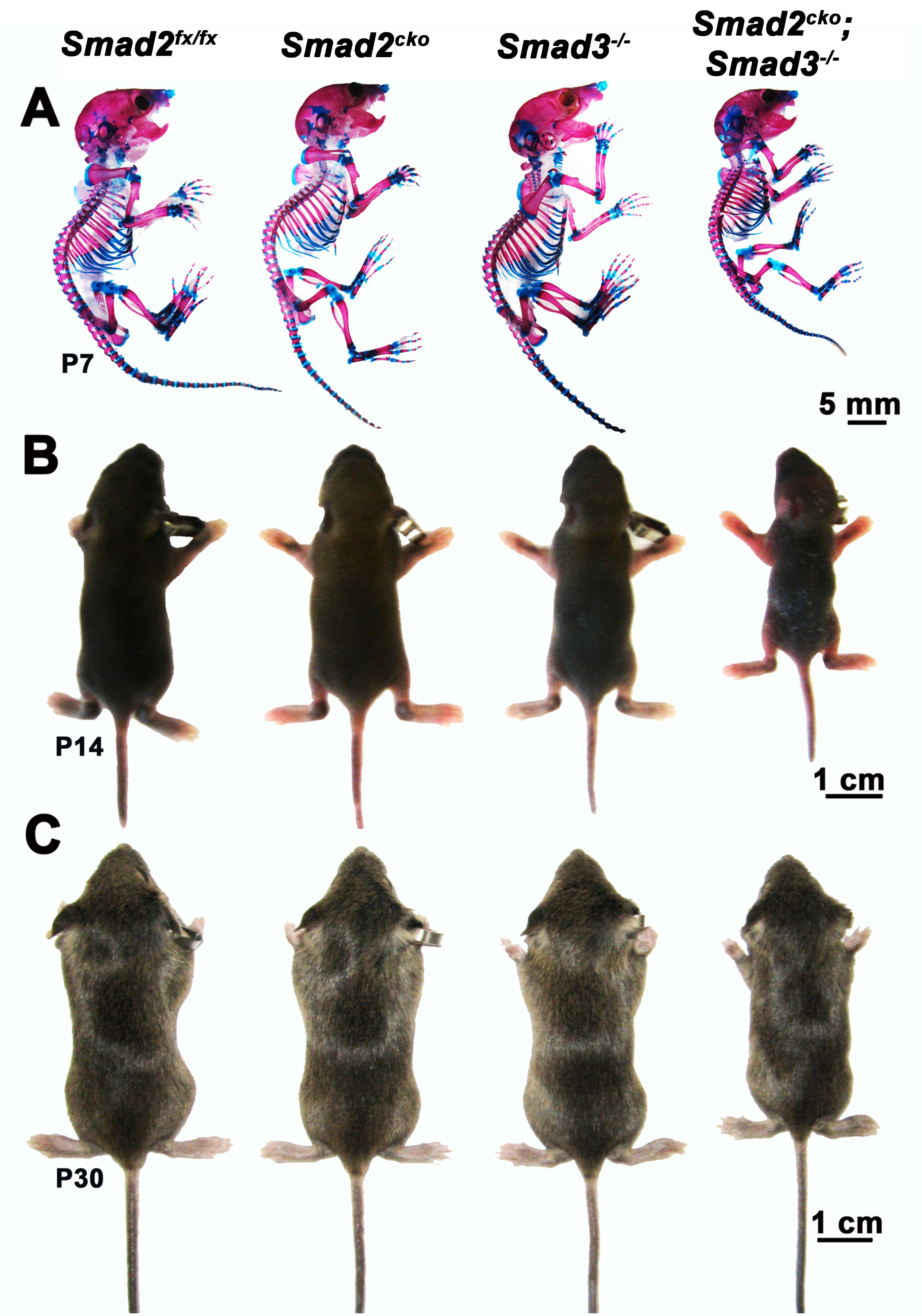

Supplement: S3 Fig — (A) Cleared skeletal preparations of P7 mice showing decreased axial length in Smad2/3 (Smad2CKO;Smad3-/-) double mutants. (B and C) P14 and P30 littermates, showing that Smad2CKO and Smad3-/- mice are not obviously smaller than control Smad2fx/fx littermates, but Smad2/3 double mutants are smaller (N = 3, P<0.05). Smad2CKO = Smad2fx/fx;Col2a1Cre. (TIF) [file pgen.1006352.s003.tif]

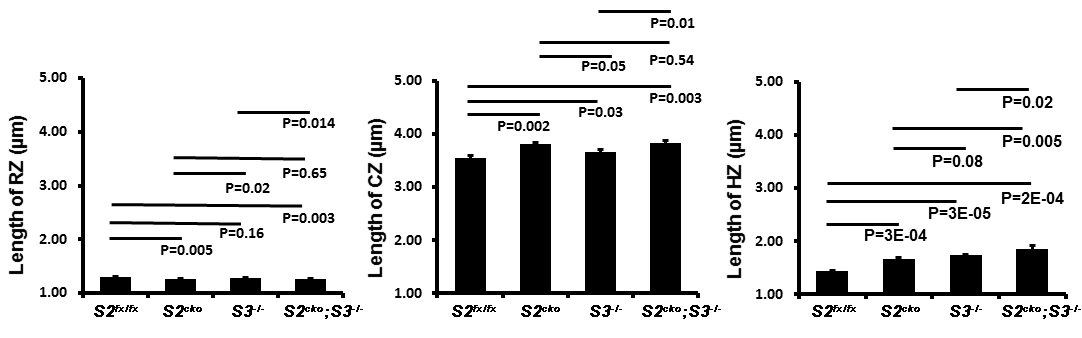

Supplement: S4 Fig — Lengths of zones were measured directly from images (n = 5) through P0 proximal tibial growth plates of littermate mice from each of 5 mice per genotype. RZ = resting chondrocyte zone, CZ = columnar chondrocyte zone, and HZ = hypertrophic chondrocyte zone. Measurements were performed by individuals blinded to genotype. Significance was established using Student's t-test. S2fx/fx = Smad2fx/fx, S2CKO = (Smad2fx/fx;Col2a1Cre), S3-/- = Smad3-/-, S2CKO;S3-/- = (Smad2fx/fx;Col2a1Cre;Smad3-/-). (TIF) [file pgen.1006352.s004.tif]

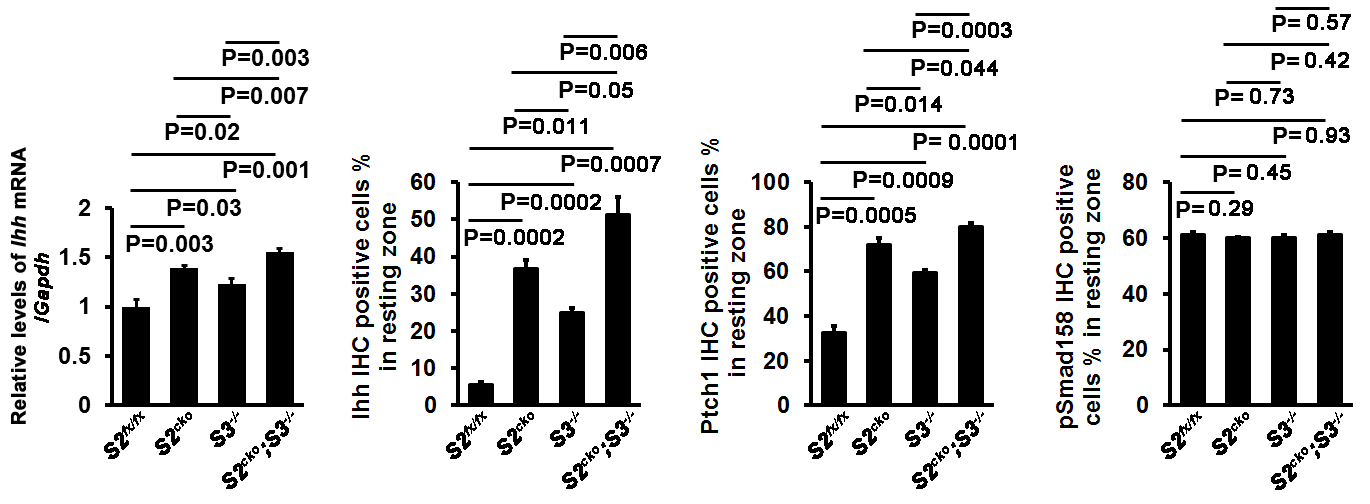

Supplement: S5 Fig — Levels of Ihh RNA were determined by quantitative real time PCR on RNA isolated from P0 growth plate cartilage. Values were normalized using Gapdh and are plotted relative to control (Smad2fx/fx) + SE (n = 3). Levels of Ihh, Ptch1 and pSmad1/5/8 proteins in resting chondrocyte zone were determinded by counting the percentage of immunohistochemistry (IHC) staining positive cells over total cells (n = 3). Significance was established using Student's t-test. S2fx/fx = Smad2fx/fx, S2CKO = (Smad2fx/fx;Col2a1Cre), S3-/- = Smad3-/-, S2CKO;S3-/- = (Smad2fx/fx;Col2a1Cre;Smad3-/-). (TIF) [file pgen.1006352.s005.tif]

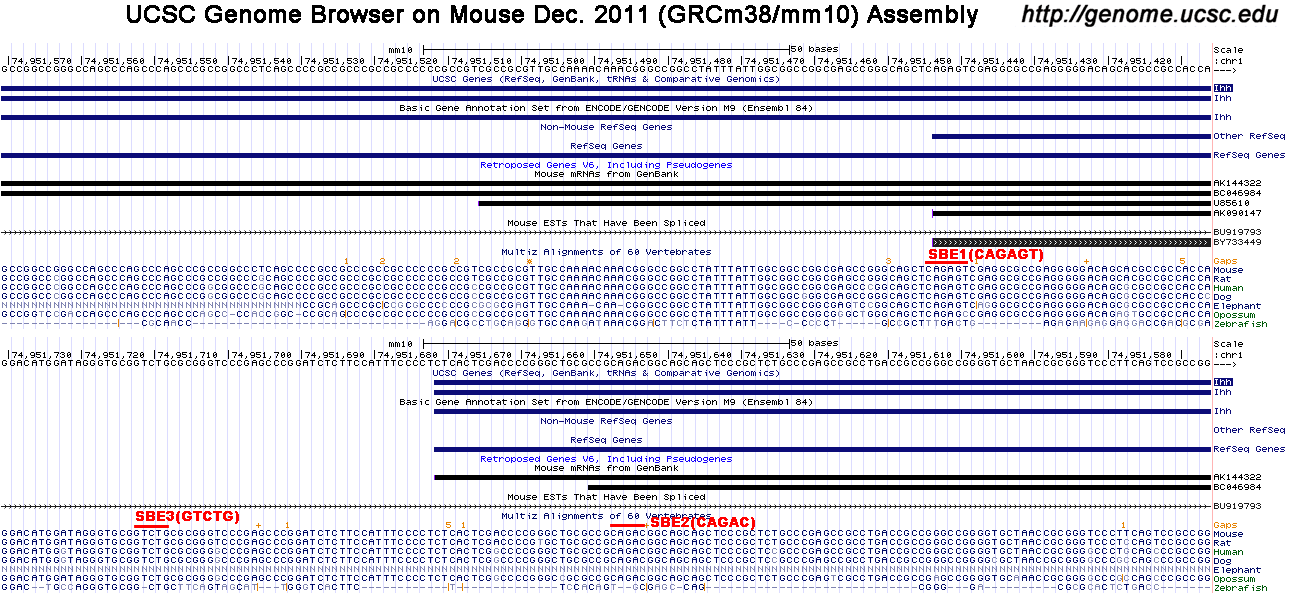

Supplement: S6 Fig — Comparative Genomic Analysis in the 5’ proximal Ihh promoter and transcription enhancer region using UCSC Genome Browser (http://genome.ucsc.edu) showed that Smad Binding Elements SBE1(CAGAGT), SBE2(CAGAC) and SBE3(GTCTG) are 100% conserved in mouse, rat, human and dog genomes. (TIF) [file pgen.1006352.s006.tif]

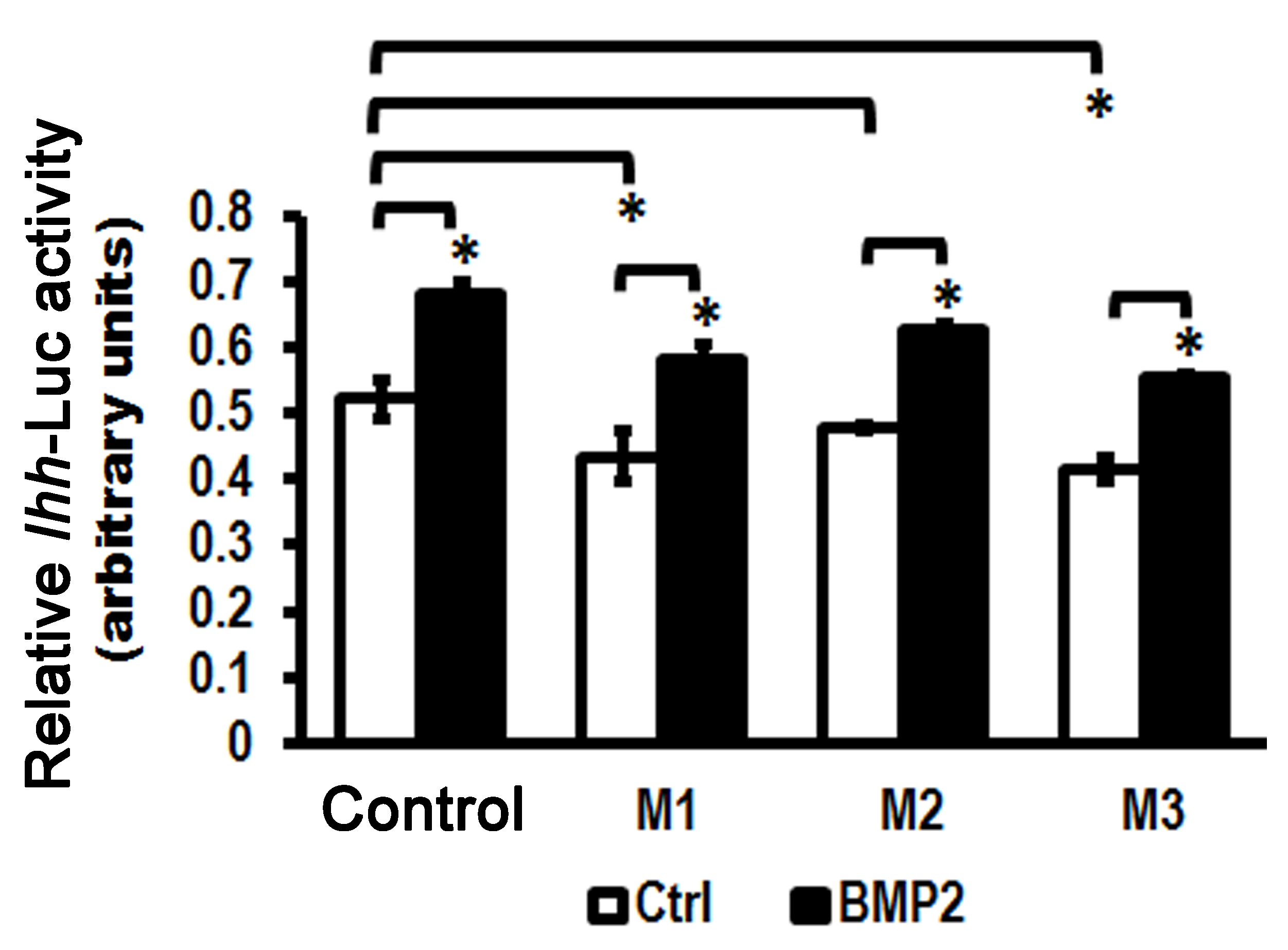

Supplement: S7 Fig — ATDC5 chondrocytes were transfected with control or mutant Ihh-promoter constructs and then treated with BMP2 (200 ng/ml) for 4 hrs. BMP2 significantly increased luciferase activities of all control and mutant constructs. There is no significant difference of BMP induction between control and mutant constructs. All experiments were performed in triplicate and repeated twice. Asterisks, p < 0.05. (TIFF) [file pgen.1006352.s007.tiff]

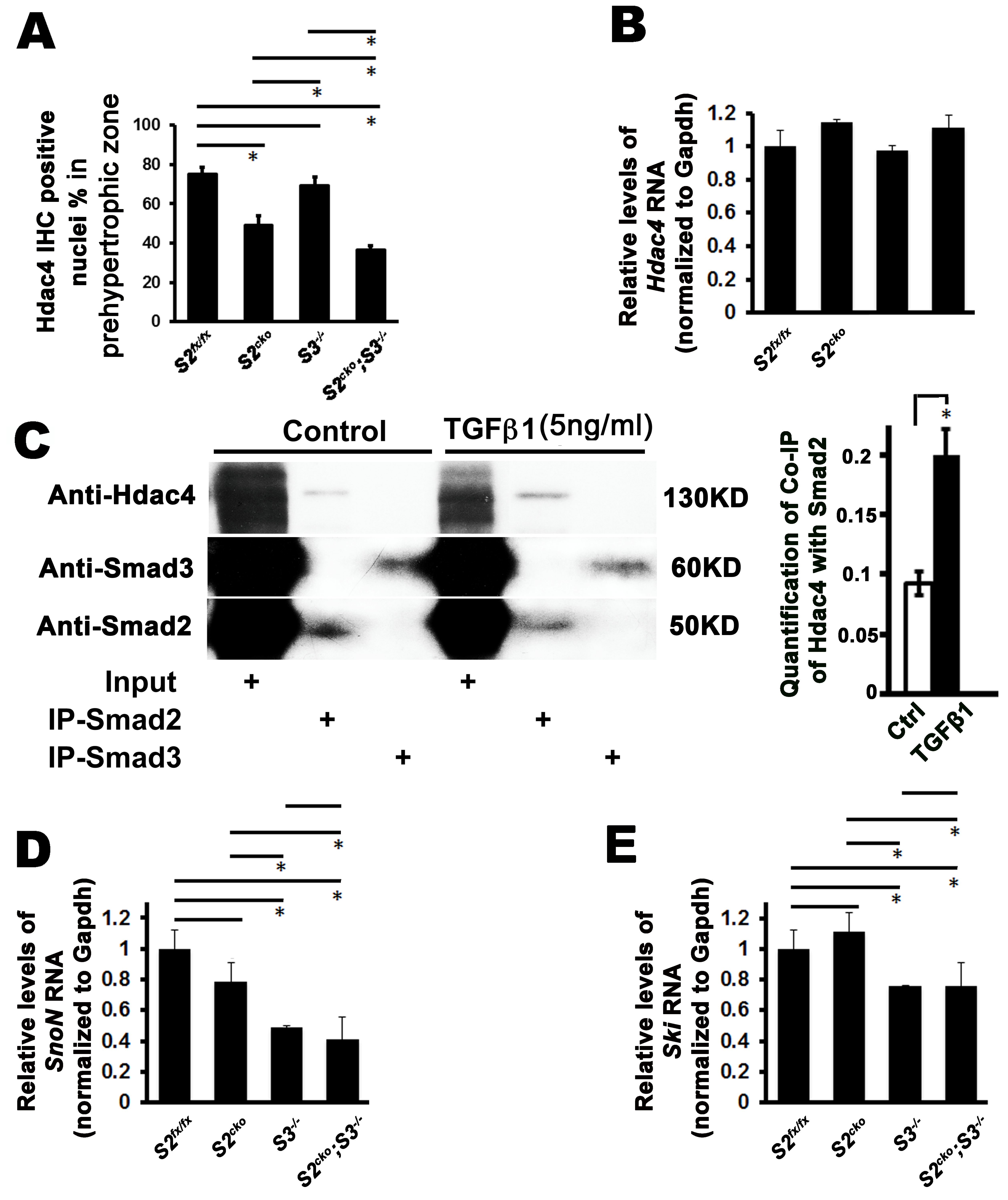

Supplement: S8 Fig — (A) Levels of Hdac4 proteins in the nucleus of prehypertrophic chondrocyte were determinded by counting the percentage of immunohistochemistry (IHC) staining-positive cells with nuclear signal over total cell numbers in the prehypertrophic zone (n = 3). (B) Quantitative real time PCR showed Hdac4 RNA level in growth plate cartilage (n = 3). (C) Co-IP analysis of Hdac4 associating with Smad2 and Smad3 in ATDC5 cells. The western blot images of Hdac4 associated with Smad2 were quantified using Adobe Photoshop CS3. The value for TGFβ1 treatment is expressed relative to control. (D-E) Quantitative real time PCR showed SnoN and Ski RNA level in growth plate cartilages. Values in B, D-E were normalized using Gapdh and are plotted relative to control (Smad2fx/fx) + SE (n = 3). Significance was established using Student's t-test. Asterisk in A, D-E, p < 0.05. S2fx/fx = Smad2fx/fx, S2CKO = (Smad2fx/fx;Col2a1Cre), S3-/- = Smad3-/-, S2CKO;S3-/- = (Smad2fx/fx;Col2a1Cre;Smad3-/-). (TIF) [file pgen.1006352.s008.tif]

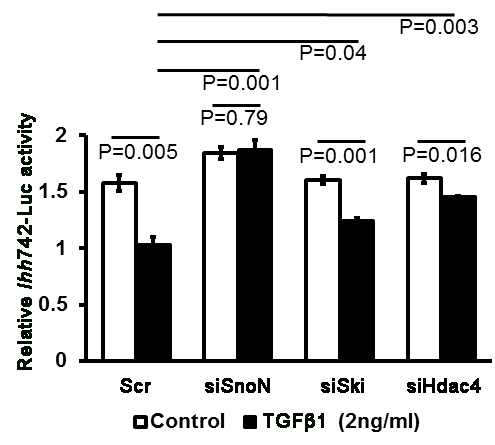

Supplement: S9 Fig — 742 bp Ihh-Luc activity in ATDC5 chondrocytes matured to prehypertrophy. Cells were treated with siRNAs against SnoN, Ski and Hdac4 expression, and then treated with TGFβ1 (2 ng/ml) for 24 hrs. Scr, scrambled siRNA control. All experiments were performed in triplicate and repeated twice. Significance was established using Student's t-test. (TIFF) [file pgen.1006352.s009.tiff]
